# Abstracts of the 2022 48th Annual NATAS Conference

**DOI:** 10.3390/polym14153104

**Published:** 2022-07-30

**Authors:** Tina Adams, Karl F. Schoch, Elizabeth Pelczar, Han Xia, Xiao Hu, Sara Reynaud, Stephen Sauerbrunn

**Affiliations:** 1 Lubrizol, Wickliffe, OH 44092, USA; tina.adams@lubrizol.com; 2 Northrop Grumman Systems Corp., Linthicum, MD 20190, USA; 3 New Jersey Department of Public Health, Ewing, NJ 08628, USA; elizabeth.pelczar@doh.nj.gov; 4 Eli Lilly and Co., Indianapolis, IN 46285, USA; hxia@lilly.com; 5 Rowan University, Glassboro, NJ 08028, USA; hu@rowan.edu; 6 Arkema, Inc., King of Prussia, PA 19406, USA; sara.reynaud@arkema.com; 7 University of Delaware, Newark, DE 19716, USA; sauerbru@udel.edu

**Keywords:** thermal analysis

## Abstract

Following the successful first-ever virtual NATAS Conference in 2021, we now hope that you are all dreaming of traveling again and that the 2022 NATAS Conference is on your list. The venue will be the historic Case Western Reserve University (CSWU) campus in downtown Cleveland. We are looking forward to seeing you in person, beginning with a welcome reception on Monday, August 1st in the beautiful Havorka Auditorium along with our vendor exhibition. Then, the week will be full of concurrent lecture sessions plus the prestigious NATAS Short Course in thermal analysis. The room block will be at the Courtyard by Marriott University Circle at a rate of USD 165 per night. We thank them for their flexibility during the various stages of this pandemic.

## 1. Introduction

The North American Thermal Analysis Society is a long-standing organization that offers scientists and practitioners the opportunity to explore the frontiers of thermal analysis, rheology, and materials characterization. Membership ensures that you have contact information for all your NATAS colleagues throughout the year and that you get the best registration rates for the annual conference. After our experience of holding a virtual conference in 2021, we are excited to move forward in 2022 with a return-to-in-person NATAS Conference at the historic Case Western Reserve University in Cleveland, OH.

Presentations and posters by renowned scientists and graduate students set the stage for excellent discussions and provide an ideal environment to learn about state-of-the-art techniques and exciting new developments in thermal analysis and materials research. Our vendors are an exemplary group well-versed in the needs of academics. You will be able to receive personalized attention and connect with providers of instrumentation and software related to thermal analysis in every way imaginable. Additionally, the NATAS Short Course is a professional-grade service and an attraction you will not want to miss.

See the 2022 conference page for more information: https://www.natasinfo.org/site_home.cfm (accessed on 1 July 2022).

## 2. Plenary Lectures

### 2.1. Promoting Accidental Discoveries and Impacting $MM Business via Thermal Analysis

KhannaYashInnoPlast Solutions*Correspondence: yash.khanna@innoplastsolutions.com

**Abstract:** Conventional wisdom is that a typical analytical laboratory should perform functions such as quality control, analytical services, and method development. However, our experience has revealed yet another facet of an analytical laboratory; that is, the creation of technologically significant concepts. While such a statement can be true for various analytical techniques, this presentation will deal with seven case histories originating from thermo-rheological techniques: (1) the development of a process to produce random copolymers of nylon 6/nylon 66 from a mixture of their homopolymers; (2) the discovery of reactor-induced memory in nylons; (3) the development of an annealing scheme to rapidly produce near-equilibrium crystals; (4) the peculiar dimensional behavior of poly(chlorotrifluoroethylene) and its technological impact; (5) super-miscible blends of nylons; (6) the peculiar melt rheology of ultra-dry nylons and its impact on industrial processing; and (7) a demonstration of the surface chemistry of stearic acid coted calcium carbonate. We claim these conceptual discoveries to be the first in the 40–60 years of existence of these materials.

### 2.2. Critical Temperature of Aviation Fuels by HPDSC

SauerbrunnSteveUniversity of Delaware—CCM*Correspondence: sauerbru@udel.edu

**Abstract:** The critical temperature is known for many pure substances, but this is not the case for organic mixtures. Jet fuel and aviation gasoline are two such mixtures where the critical temperature requires determination for flight system engineering. If the fuel is at critical pressure and temperature (supercritical fluid) there is no heat of vaporization (HOV). In this case, there is no drop in temperature as the fuel is injected into the combustion chamber. This will improve the fuel efficiency of the engine. The high-pressure DSC was used to determine the critical temperature. The HPDSC was set up so that it would ramp pressure while keeping the sample at a quasi-isothermal temperature. The sample then evaporates as the pressure decreases, giving the HOV at that temperature. Several of these experiments were undertaken at a range of temperatures. The HOV decreases as the QI temperature increases. The HOV versus temperature is then extrapolated to zero HOV. This temperature is the critical temperature for the mixture. The HPDSC experimental setup will be described in detail. The results for several commercial and military jet fuels will be shown together with aviation gasoline as used in light aircraft.
**Funding:** This work was funded by the CRC Aviation Fuels Committee.

### 2.3. Thermal Analysis for Structure–Processing–Property Relations of Multi-Functional Polymer Materials

MeleBruno vanVrije Universiteit Brussel*Correspondence: bruno.van.mele@vub.be

**Abstract:** Over the course of more than 30 years, dedicated protocols were developed based on advanced thermal analysis (modulated temperature DSC, rapid heat-cool DSC, chip-based AC and fast scanning calorimetry, AFM-based thermal analysis, micro(nano)-calorimetry, DMA, dynamic rheometry, etc.) to characterize and understand the structure–processing–property relations of various multi-functional polymer systems. Some of following topics will be highlighted in this presentation:

o Reactive polymer systems
- Mechanistic modelling of the irreversible cure kinetics of organic thermosetting resins, and the influence of chemo-rheology (gelation, vitrification) on the reaction process
- Experimental study of inorganic polymer networks (geopolymers)
- Reaction-induced phase separation
o Polymer blends and polymer solutions
- Modulated temperature DSC to study phase separation and mixing
o Thermoplastic starches (biodegradable polymers)
o Nano-composites
- Modulated temperature DSC to study the glass transition and crystallization at the fiber/matrix interphase in nano-composites
o Polymers for controlled drug delivery (biomedical applications)
- Rheological study of injectable peptide carriers for drug release
o Thin layers and coatings
- Fast-scanning calorimetry approaches including rapid heat-cool DSC (RHC) and ultrafast-scanning chip calorimetry. Application to metastable nano-morphology development in thin films
- AFM-based spatially resolved thermal analysis and AC chip calorimetry of smart polymer surfaces
- Thermal analysis of atmospheric plasma deposited coatings
o Conjugated polymer or small molecule mixtures with fullerenes for organic photovoltaic solar cells (OPV) and photodetectors (NIR OPD) (energy applications)
- RHC study of the thermal transitions and nano-morphology of donor/acceptor mixtures (push-pull conjugated polymers, small molecules).
- The state diagram of P3HT:PC61BM mixtures with emphasis on structural disorder and its importance for the stability of organic solar cells
- Characterization of eutectic behavior in OPV mixtures
o Self-healing dynamic polymer networks (sustainable materials, protective coatings)
- Development of self-healing networks with dynamic covalent bonding based on thermo-reversible Diels–Alder (DA) chemistry
- DA reaction kinetics and thermodynamics, thermo-mechanical behavior of the networks
- Diffusion- and mobility-controlled self-healing thermosets.

## 3. Session: Thermo-Physical Properties

### 3.1. Thermal Expansion and Surface Roughness Study of Pellet to Cladding Interaction in CFR600 Design

PengYanChina Institute of Atomic Energy
pengyan@ciae.ac.cn

**Abstract:** CFR600 is a pool-type sodium-cooled fast reactor. Its thermal power is about 1500MW and its electrical power is about 600 MW. The fuel material is MOX of 30 wt% PuO2 and 70 wt% UO2, and the cladding is 15–20% CW15-15Ti [1]. The irradiation deformations of the pellet and cladding can worsen the heating transfer through the rods, the assemblies, and the reactor core. So, research into the pellet to cladding interaction (PCI) phenomena is an important factor in CFR600 design. PCI includes mechanical and chemical interactions. Mechanical PCI is caused by the strain which is formed by the deformations of irradiations, and chemical PCI involves the diffusion processes of complex multi-phases [2]. These interactions can greatly change the roughness conditions of the fuel and the cladding surfaces, which directly affect the critical heat flux (CHF) along the non-uniformed heating flow channels [3,4]. In this paper, based on the versus relationships between the cladding diameter and the axial position along the fuel assembly, we tried to use the thermal measurements [5,6] to confirm the surface roughness changes of the fuel and the cladding due to the different thermal expansion coefficients which are changed with the increase in CFR600 burnup.

**References** 

1. Chen HUANG. CEFR joint materials irradiation and testing plan. CEFR Platform for R&D International Cooperation on Fast Reactor. Beijing: 2013.
2. R.B. Baker. Calibration of a fuel to cladding gap conductance model for fast reactor fuel pins. HEDL-TME 77-86 UC-79. 1978.
3. Jiyang YU, Baoshan JIA. Reactor thermal hydraulics. Tsinghua University Press. Beijing: 2003. pp.174-185.
4. Buxuan WANG. Research on thermal sciences. China Machine Press. Beijing: 2002. pp.148-153.
5. ASTM E1530. Standard test method for evaluating the resistance to thermal transmission of materials by the guarded heat flow meter technique. Annual Book of ASTM Standards. 2017; Volume 14.05:336-344.
6. David L. Clark, David A. Geeson, Robert J. Hanrahan. Plutonium handbook. American Nuclear Society. La Grange Park, Illinois: 2019. pp.3155-3165.”

### 3.2. Effects of Adding Phosphorus-Containing Ionic Liquid on Flammability and Thermal Stability of Paraffin Wax

ShuChi-Min*LinYu-ChengDepartment of Safety, Health, and Environmental Engineering, National Yunlin University of Science and Technology*Correspondence: shucm@yuntech.edu.tw

**Abstract:** Paraffin, a hydrocarbon mixture extracted from petroleum, is often used as a phase change material for many products. However, there have been many fire accidents relating to paraffin flammability which resulted in significant loss. The risk prevention for fire hazards relating to paraffin can be developed based on two aspects: the first is reducing the probability of ignition and high temperature contact, and the second is increasing the flame retardant properties of paraffin. Among the substances used in these processes, ionic liquids (ILs) have good thermophysical properties. In recent years, they have also been used to improve the thermophysical properties of other substances. Phosphorus-containing ILs have the most outstanding flame-retardant effect. In this study, two phosphorus-containing ILs, 1-butyl-3-methylimidazolium dibutylphosphate ([Bmim][DBP]) and 1-butyl-3-methylimidazolium hexafluorophosphate ([Bmim][PF_6_]), were selected as additives to modify the flame retardancy of paraffin. The above-mentioned ILs and diatomaceous earth were added into the paraffin then solidified to obtain the flame-retardant paraffin. Paraffin mixed with diatomaceous earth had the highest exothermic heat flow (10.23 W/g) in all specimens tested by a simultaneous thermogravimetric analyzer (STA). In contrast, paraffin mixing with diatomaceous and ILs restrained the heat release rate (about 4.42—4.51 W/g) in a high-temperature environment. The initial temperature of the pyrolysis zone (Ti) and the final temperature of the pyrolysis zone (Tf) of paraffin are significantly delayed in the thermogravimetric analysis after containing ILs. The decomposed gas production of carbon dioxide (O=C=O) and alkanes (C-H) was observed by means of thermogravimetric analysis coupled with Fourier transform infrared spectroscopy (TGA-FTIR). Finally, thermokinetics was used to evaluate the thermal stabilities of different compositions of flame-retardant paraffin samples. When the conversion rate is between 0.10 and 0.50, the apparent activation energy (Ea) of the paraffin wax tends to decrease gradually. Through the above analyses, paraffin containing 8% [Bmim][DBP] or 4% [Bmim][PF_6_] had the best flame retardancy.

### 3.3. Effects of Lycopodium, Corn Starch, and Coal Dust Dispersion Pressure on Minimum Ignition Temperature of Dust Cloud

ShuChi-Min*SyueGuo GanDepartment of Safety, Health, and Environmental Engineering, National Yunlin University of Science and Technology*Correspondence: shucm@yuntech.edu.tw

**Abstract:** In recent years, with the progress of the industry, the potential explosion and combustion hazards caused by dust have been received attention around the world. Many dust disasters occur around the world every year, caused by dusts such as mineral dust, wood dust, food dust, metal dust, plastic dust, etc. Among them, the minimum ignition temperature of a dust cloud (MITC) is a crucial parameter of dust hazards, and the analytical method follows IEC 61241-2-1: 1994, which specifies the sample mass and dispersion pressure during the test. In recent years, many studies have pointed out the MITC of various dusts and tested their combustion characteristics, but all of them are based on the dust mass as a variable factor for analysis, and few studies have discussed the air dispersion pressure. This study used samples of Australian coal dust, commodity corn starch, and lycopodium to conduct MIT tests and observed the influence of the following dispersion pressures: 0.05, 0.1, 0.2, 0.3, and 0.5 barg. The MITC test was carried out using a Godbert–Greenwald furnace (G–G furnace), and we compared the flame propagation speed in each dust under different dispersion pressures with a high-speed camera. In addition, we compared the relationship between the inlet pressure and the mass of the dust sample before and after being blown out in the G–G furnace without heating, and finally explored the minimum explosion concentration and intake pressure gravimetric to compare the flammable limit concentration results. The results show that the lower the dispersion pressure, the higher the MITC in corn starch and lycopodium dust, and there is no prominent difference in pulverized coal dust. Therefore, the dispersion pressure will indeed affect the results of MITC. It is recommended to set it above 0.3 barg to ensure that the correct inherent hazard parameters are obtained.

### 3.4. Analysis of Increasing Glass Transition Behavior of Polybenzoxazine during Pyrolytic Conversion

SauerbrunnSteve*MuhammedFaheemUniversity of Delaware—CCM*Correspondence: sauerbru@udel.edu

**Abstract:** Polybenzoxazine thermoset resins have been utilized as a carbon matrix precursor for carbon/carbon composites due to their high char yield post-pyrolysis. During pyrolysis, the resin is converted to amorphous carbon through a series of decomposition mechanisms and molecular reorientation steps. Based on the non-isothermal kinetics of pyrolytic conversion, thermal cycles consisting of variable ramp rates and isothermal dwell times were conducted using a Mettler coupled TGA/DSC, under a constant flow rate of nitrogen in the range of 25–1000 °C, to condition samples to varying extents of conversion (alpha). The glass transition temperature (TG) of the samples were measured as a function of alpha and increased in an exponential fashion from 138 to 350 °C at 80% conversion. This was expected, as it is believed that the TG exists as an intermediate between that of the resin and of amorphous carbon. This is due to the polymer molecule cleaving heteroatoms, and undergoing aromatization during pyrolysis [1], which has been shown to increase the TG of other polymer systems [2]. In performing the analysis, a relationship between TG and alpha was gleaned, and could be represented by a modification of Pochan’s logarithmic expression for polymer blends [3].

**References** 

1. J. Hacaloglu, T. Uyar, and H. Ishida, “Thermal degradation mechanisms of polybenzoxazines,” in Handbook of Benzoxazine Resins, no. January, Else, Ed. Amsterdam: Elsevier, 2011, pp. 287–305.
2. H. T. H. Nguyen, P. Qi, M. Rostangno, A. Feteha, and S. A. Miller, “The quest for high glass transition temperature bioplastics,” vol. 6, no. 20, 2018, doi: https://doi.org/10.1039/C8TA00377G.
3. A. Mushtaq, H. Bin Mukhtar, and A. M. Shariff, “Effect of Glass Transition Temperature in Enhanced Polymeric Blend Membranes,” Procedia Eng., vol. 148, pp. 11–17, 2016, doi: 10.1016/j.proeng.2016.06.448.

## 4. Session: Rheology

### 4.1. Linear—Nonlinear Transition in the Dynamics of Entangled DNA

BanikSourya^1^McKennaGregory^2^1Texas Tech University2North Carolina State University
gbmckenna@ncsu.edu

**Abstract:** DNA is often taken as a model linear polymer and has been found to exhibit dynamic viscoelastic properties that are similar to those of similarly entangled synthetic polymers. However, there are relatively few reports on the linear to nonlinear transition in the viscoelastic response of entangled DNA solutions. Here, we report results from a study of the dynamic moduli G′ and G″ for entangled DNA solutions. Surprisingly, it is found that ideally monodisperse lambda phage DNA (M = 48.5 kbp = 3.15 × 10^7^ g/mol; PDI = 1.0) solutions (1.6 mg/mL and 3.2 mg/mL) exhibit nonlinearity in strain sweep mode at strain amplitudes far smaller than those observed in synthetic polymers. At the same time, it was found that a polydisperse calf thymus DNA (Mw = 23 kbp = 1.49 × 10^7^ g/mol; PDI = 2.1) does not exhibit extreme strain sensitivity. This led to the hypothesis that the ideal monodisperse nature of the lambda DNA is responsible for such behavior. To test the hypothesis, we mixed samples of the monodisperse lambda DNA with the polydisperse calf thymus DNA and found that the magnitude of the strain at which nonlinearity commenced increased with the DNA solution’s polydispersity. For the entangled lambda DNA with PDI = 1.0, the onset of nonlinear response occurred at a strain gamma less than 2%.

The authors thank the National Science Foundation under grant CBET 1603943 and the J.R. Bradford endowment at Texas Tech University, each for their partial support of this project.

### 4.2. Rheology-Combined Neutron Techniques in Multi-Functional Material Development

NguyenNgocApplied Research Institute, University of Illinois at Urbana-ChampaignCorrespondence: nanguyen@illinois.edu

**Abstract:** In this talk I will present perspectives around using in situ rheo-neutron reflectometry, small angle neutron scattering, and wide-angle X-ray scattering in the study of a five-dimensional material demonstrating excellent shape memory effects, self-healing responsiveness, and good 3D printability for additive manufacturing applications. Detailed morphological, rheological, thermal, and mechanical characteristics of the multi-functional material will be discussed. This material was developed using a low-valued renewable material containing abundant functional groups such as the dopamine structure found in marine mussels.

### 4.3. Stress Activated Constraints in Dense Suspension Rheology

SinghAbhinendraJaegerHeinrich M.PabloJuan J deUniversity of Chicago*Correspondence: asingh.iitkgp@gmail.com

**Abstract:** Both heterogeneous nucleation and flow-induced entropy reduction are well-known factors that accelerate polymer crystallization. However, the interplay between nucleation and flow-induced acceleration is still poorly understood. This work investigates the nucleating effect of carbon nanotubes (CNT) on both the quiescent and flow-induced crystallization kinetics of polyamide 66 (PA 66). The quiescent crystallization study indicates that CNT acts as a powerful nucleant, as suggested by the fact that the critical cooling rate to bypass crystallization and create the amorphous glassy state changes from 1000 K/s in PA 66 neat resin to a rate faster than 4000 K/s in the PA 66 nanocomposites. The flow-induced crystallization study indicates that PA 66’s onset crystallization time and morphology depend on the shear work introduced by rotational rheometry. A combined acceleration effect from CNT nucleants and FIC persists when the CNT loading is below the saturation limit. However, if CNT loading meets the saturation limit, specific shear work shows no impact on the crystallization time, providing evidence that the role of the FIC acceleration effect no longer exists when heterogeneous nucleation acceleration dominates the crystallization of PA 66.

### 4.4. Transient Rheology and Anisotropic Structure of Attractive Colloidal Suspensions Studied with Orthogonal Superposition

WangYilin*EwoldtRandyUniversity of Illinois at Urbana Champaign*Correspondence: yilin7@illinois.edu

**Abstract:** This study resolves a long-existing ambiguity of experimentally observed transient stress growth-then-decay of carbon black (CB) colloidal suspensions under a constant applied shear rate [1] by combining step-down shear flow with orthogonal superposition (OSP). We reveal that the short-term stress recovery observed in CB suspensions is related to thixotropy, which is also called aging (at rest) and rejuvenation (under flow), while the long-term stress decay is associated with anti-thixotropy, rather than viscoelasticity, by superimposing an oscillatory flow orthogonal into the shear flow on a rotational rheometer, to probe viscoelastic moduli. The orthogonal elastic modulus is present, showing this two-timescale response, which demonstrates that this response involves shear-induced structuring that stores elastic energy. We further show a mechanical anisotropy in the CB suspension under shear using OSP. Therefore, a microstructural schematic is proposed, considering qualitatively thixotropic structure build-up, anti-thixotropic densification, and anisotropic log rolling. Our observation for these CB suspensions is outside the standard paradigm of thixotropic structure–parameter models, and the elastic response provides us with new insights into this two-timescale thixotropy of CB suspensions.

**References** 

1. New insights on carbon black suspension rheology—anisotropic thixotropy and anti-thixotropy, Y. Wang and R. H. Ewoldt, Preprint; doi:10.48550/arxiv.2202.05772

### 4.5. Progress in the Determination of the Complex Poisson’s Ratio Using Combined Torsional-Axial Measurements on a MultiDrive Rheometer Platform

ShettyAbhishek^1^*AgudoJose Alberto Rodriguez^2^HaeberleJan^2^ArnoldGunther^2^GiehlChristopher^2^Anton Paar USAAntor Paar Germany*Correspondence: abhi.shetty@anton-paar.com

**Abstract:** The lateral contraction of a material when stressing the material in the axial direction is described by the Poisson’s ratio [1]. In the case of viscoelastic materials, such as polymers, this parameter is a function of temperature and excitation frequency, when measured in oscillatory mode, and important for, e.g., structural mechanics simulations [2]. Methods to determine the viscoelastic Poisson’s ratio are manifold and can be classified in direct methods, which directly measure the change in the specimen dimension, and indirect methods from which the measurement of two moduli such as shear modulus and Young’s modulus seems to be the most effective [3]. A new measuring device concept is introduced which combines an electronically commutated rotational top drive and a moving magnet linear drive as the bottom drive to enable torsional and axial measurements on one single device. In this contribution, measurements on both cylindrical and rectangular specimens are presented in order to determine the viscoelastic Poisson’s ratio of different solid polymers. Using a linear and a rotational measuring drive in one instrument enables the determination of complex shear modulus |G*| as well as the complex Young’s modulus |E*| on a single specimen in a continuous measurement run. Consecutive frequency sweeps in both torsion and tension deformation modes were performed to obtain the temperature- and frequency-dependent viscoelastic Poisson’s ratio. A suite of polymers ranging from amorphous (PMMA, PC), thermoplastic polyurethane (TPU) to thermosets (Epoxy) were studied. For amorphous polymers, a nonmonotonic increase in the complex Poisson’s ratio with temperature was found when approaching the glass transition temperature (Tg) [4]. For the other polymers, a monotonic increase in the complex Poisson’s ratio with temperature was observed around Tg [5]. The suitability of the method is further examined and discussed by comparing the experimental results on several polymers with literature data.

**References** 

1. Poisson, S. D. (1827) Note sur l’extension des fils et des plaques elastiques. Annales de Chimie et de Physique 36: 384–385.
2. Tschoegl, N.W., Knauss, W. G., Emri, I. (2002) Poisson’s Ratio in linear viscoelasticity –a critical review. Mechanics of Time-Dependent Materials 6: 3-51.
3. Pritz, T. (2000) Measurement methods of complex Poisson’s ratio of viscoelastic materials. Applied Acoustics 60: 279-292.
4. Grassia, L., D’Amore, A., Simon, S. L. (2010) On the viscoelastic Poisson’s ratio in amorphous polymers. Journal of Rheology 54: 1009.
5. Pandini, S., Pegoretti, A.: Time and temperature effects on Poisson’s ratio of poly (butylene terephthalate). Polymer Letters 5 (2011) 685-697.

### 4.6. Thermodynamics, Dynamics and Rheology of Imidazolium-Based Ionic Liquids in Bulk and under Confinement Conditions

LazarenkoDaria*KhabazFardinThe University of Akron*Correspondence: dsl32@uakron.edu

**Abstract:** Ionic liquids (ILs) are molten salts known for their low vapor pressure and promising thermo-rheological behavior. ILs have been proposed as potential candidates for enhancing the tribological performance of conventional lubricants and making them more environmentally friendly. In this work, the thermodynamics, dynamics, and rheology of various imidazolium-based ILs are studied utilizing all-atom molecular dynamics (MD). The simulations are used to investigate the bulk volumetric properties of ILs such as density and coefficient of thermal expansion. The diffusion coefficients are explored to measure the dynamic properties of the system as a function of temperature. Non-equilibrium MD is utilized to perform shear simulations. The shear viscosity of the ILs is calculated and a Newtonian plateau and shear-thinning behavior are identified in the systems. The viscoelastic behavior of ILs is evaluated from the dynamic modulus calculations. The time–temperature superposition (TTS) principle is used to collapse the mean squared displacement, shear viscosity, and dynamic moduli of cations and anions onto universal curves by applying a single set of shift factors. TTS allows the bridging of the timescale between simulations and experiments. A metal surface is then introduced to confine the ILs. The nanostructure at the solid–liquid interface directly influences the tribology of the lubricant. To understand the behavior of molecules at the interface and under applied pressure, density correlations are calculated along with the orientation of molecules on the solid surface. The velocity gradient of the lubricant under nanoconfinement is characterized by studying the induced shear flow generated by the motion of the explicit surfaces. The stress response and flow behavior of the liquid are tracked to understand the slip mechanism at the solid–liquid interface. Lastly, the viscosity, normal forces, degree of confinement, and flow rate are connected to the friction coefficient of the system by defining a modified Hersey number. The outputs from this project will allow the inverse design of high-performance IL-based lubricants for a non-toxic solution to energy loss due to friction and wear.

### 4.7. Rheological Properties of Thermal Interface Materials

SchochKarlJr.*PanackalPhilipBrockiAmandaNorthrop Grumman Corporation*Correspondence: k.eric.schoch@ngc.com

**Abstract:** Thermal interface materials (TIMs) perform a vital function in electronic assemblies by ensuring efficient heat transfer away from heat-generating components. There are several types of TIMs, including pastes, phase change materials, adhesives, cure-in-place gap fillers, and greases. Pastes are the subject of this paper. They have the desirable properties of accommodating a range of bondlines, being easy to dispense and rework, when necessary, and tolerating a difference in thermal expansion behavior between adherends. These materials typically have a thermally conductive filler dispersed in a polymer matrix. In this paper, we discuss the how the rheology of these highly filled pastes affects the assembly of the electronics hardware. For example, the effective modulus of the material is dependent on the rate of applied deformation. Therefore, controlling the deformation rate during assembly is important in order to avoid damaging the components by applying excessive force. The force applied to the assembly is also affected by the shape factor (surface area/perimeter area ratio), which needs to be considered. These pastes can exhibit viscosity recovery time in the order of hours, which also affects the behavior of these materials during assembly of the hardware. Examples of each of these phenomena will be provided to illustrate the importance of considering rheological properties when choosing TIMs and developing processes for assembling hardware.

### 4.8. Glass Transition and Entanglement in Semiflexible Conjugated Polymers

ColbyRalph*FentonM.XieRenxuanGomezEnriquePenn State University*Correspondence: rhc5@psu.edu

**Abstract:** Polymers with conjugated backbones and flexible side chains are semiflexible and exhibit two glass transitions, one for the conjugated backbone and a lower Tg for the side chains. Both glass transitions are broadened, since the side chains are covalently attached to the backbones, and are challenging to detect by standard calorimetry methods (i.e., DSC). By compression molding in a glove box, it is possible to measure the linear viscoelastic response (LVE) of conjugated polymers from the lower Tg up to roughly 300 °C. The two glass transitions are detected in LVE temperature sweeps at 1 rad/s as local maxima in the loss modulus G″ and the width of these transitions is reflected in how much higher in temperature tan δ = G″/G′ exhibits a local maximum [1]. A simple model is presented to predict the backbone glass transition from the volume fraction of the backbone and the molecular characteristics of the backbone [2], enabling molecular design of conjugated polymers for flexible electronics with Tg below ambient. Many of the conjugated polymers with more rigid backbones exhibit liquid crystalline phases above their melting temperature. The ones with a nematic phase are very easy to detect in the LVE temperature sweeps, as the nematic A isotropic transition is easily seen as a roughly 10 K window where viscosity actually increases as temperature is raised [3,4] (which is the biphase), whereas this weakly first order transition is virtually undetectable by DSC. In the isotropic phase at high temperature, the plateau modulus is measured by constructing an LVE master curve, and with the molecular weight distribution, this is fit to a molecular LVE model based on reptation dynamics, originally developed for more flexible polymers. The Kuhn length is determined either by small-angle neutron scattering in solution or by calculation using the bond angles and lengths via the freely rotating chain model. For semiflexible polymers, these two methods agree nicely and the plateau modulus is correlated with backbone stiffness, allowing prediction by estimating the Kuhn length [4]. This is very powerful, since the plateau modulus (number density of entanglements) is vital for understanding the semicrystalline modulus of conjugated polymers that have Tg below room temperature, which is needed for flexible electronics [5].

**References** 

1. R. Xie, Y. Lee, M. P. Aplan, N. J. Caggiano, C. Muller, R. H. Colby and E. D. Gomez, Glass Transition Temperature of Conjugated Polymers by Oscillatory Shear Rheometry, Macromolecules 50, 5146 (2017).
2. R. Xie, A. R. Weisen, Y. Lee, M. A. Aplan, A. M. Fenton, A. E. Masucci, F. Kempe, M. Sommer, C. W. Pester, R. H. Colby and E. D. Gomez, Glass Transition Temperature from the Chemical Structure of Conjugated Polymers, Nat. Comm. 11, 893 (2020).
3. R. Xie, M. P. Aplan, N. J. Caggiano, A. R. Weisen, T. Su, C. Muller, M. Segad, R. H. Colby and E. D. Gomez, Local Chain Alignment via Nematic Ordering Reduces Chain Entanglement in Conjugated Polymers, Macromolecules 51, 10271 (2018).
4. A. M. Fenton, R. Xie, M. P. Aplan, Y. Lee, M. G. Gill, R. Fair, F. Kempe, M. Sommer, C. R. Snyder, E. D. Gomez and R. H. Colby, Predicting the plateau modulus from molecular parameters of conjugated polymers, ACS Central Science 8, 268 (2022).
5. R. Xie, R. H. Colby and E. D. Gomez, Connecting the Mechanical and Conductive Properties of Conjugated Polymers, Advanced Electronic Materials, 1700356 (2017).

### 4.9. Effect of Temperature on Wormlike Micelle Rheology at High Shear Rates

SalipantePaul*HudsonStevenNational Institute of Standards and Technology*Correspondence: paul.salipante@nist.gov

**Abstract:** Wormlike micelles are polymer-like aggregates that self-assemble from amphiphilic surfactant molecules. The length and rheology of these micelles are sensitive to temperature due to the change in strength of association relative to thermal energy. Solutions of wormlike micelles are typically strongly shear thinning due to the change in structure under shear, and at high shear rates a decrease in micelle length is anticipated. Using capillary and microfluidic rheology, we investigate the effect of temperature on the high shear rate rheology of the wormlike micelles. A small-volume microcapillary rheometer within a temperature-controlled box is used to measure shear rates in the range of 100 s^−1^ to over 105 s^−1^. The relaxation time of the fluids is measured by tracking the decay of birefringence after rapid flow cessation in a microfluidic channel. We compare solutions of different surfactant concentrations and compositions at various temperatures to infer the change in structure under high shear rates.

### 4.10. Incorporating Rheo-Combinatorial Techniques to Overcome Polymer Processing Challenges

EickhoffJames*ShettyAbhishekAnton Paar USA*Correspondence: james.eickhoff@anton-paar.com

**Abstract:** Polymers are used in a wide range of applications, from both an industrial perspective as well as one’s day to day life. Implementing both mechanical and thermal analysis techniques helps to ensure exceptional performance, not only from a processing point of view, but also from an end use product standpoint. A series of polyethylene polymers (LDPE, HDPE, etc.,) as well as pressure sensitive adhesives were explored in this study. These samples were investigated using a range of traditional dynamic oscillatory shear and steady start up shear experiments. These tests were performed over a range of processing conditions to further evaluate the material properties. The polymers investigated showed simple thermo-rheological behavior, and time–temperature superposition (TTS) was used to extend the dynamic frequency range. The modelling of polymer processing operations typically requires the relaxation and retardation spectrum of the polymer. In this work, relaxation spectra were obtained from the TTS data as described above. Quantitative analysis of the steady start up shear data of the polymers by using the damping function is also shown. Finally, rheo-microscopy as a combinatorial technique is also looked at to analyze the crystallization behaviors of LDPE polymers and implications from a processing standpoint are discussed.

## 5. Session: Fast-Scan Techniques

### 5.1. Rapid Solidification of Al Cu Alloys Using Fast Scanning Calorimetry

NarayanLakshmi RaviHebertRainerUniversity of Connecticut
lakshmi@uconn.edu

**Abstract:** Metal alloys solidify over a range of temperatures, and both the range of temperatures over which solidification occurs and the rate at which the solid fraction forms depend on the composition of the alloy and the cooling rate. Fraction of solid curves represent the solidified fraction as a function of temperature and are an important input for material models used for simulating manufacturing processes. They can be used to predict the susceptibility to defects such as solidification cracking, the microstructure upon solidification and to simulate solidification-based manufacturing processes. This study reveals the fraction of solid curves for hypoeutectic aluminum-copper compositions during rapid cooling from the liquid state, determined using chip-based fast scanning calorimetry. The experimental methods for performing chip-based calorimetry with metallic materials are described. A method for correcting for the smearing effect using experimentally determined time constants that was used for this material is also described. The data from this experiment were used to plot an experimental kinetic phase diagram for the hypoeutectic region of the Al-Cu system. The results from this work are useful for modelling and simulating processes with metal powders.

### 5.2. Glass Forming Ability of Polyzwitterions

CebePeggy^1^ClarkAndrew^1^BiswasYajnaseni^1^TaylorMorgan^1^AsatekinAyse^1^PanzerMatthew^1^SchickChristoph^2^Tufts UniversityUniversity of Rostock
peggy.cebe@tufts.edu

**Abstract:** The glass forming ability of a series of specially synthesized polyzwitterions was studied using fast scanning calorimetry (FSC) [1]. Polyzwitterions include those based on the sulfobetaine moiety: sulfobetaine acrylate (SBA), ethyl sulfobetaine methacrylate (ESBMA), sulfobetaine vinylimidazole (SBVI), sulfobetaine 4-vinylpyridine (SB4VP), sulfobetaine methacrylate (SBMA), and sulfobetaine methacrylamide (SBMAm). FSC was used to investigate the dynamic fragility over a large range of cooling rates, 10–4000 K/s, minimizing the thermal degradation of the polyzwitterions. The rate dependence of the limiting fictive temperatures (Tf) was measured and fit to the Williams–Landel–Ferry model, from which the polyzwitterion dynamic fragility was determined for the first time. Dynamic fragility was low, ranging from 41 to 110 depending on the underlying chemical structure, which allows the classification of this series of polyzwitterions as moderate to relatively strong polymeric glass formers. Their high glass transition temperatures combined with low fragilities indicate that polyzwitterions are unique among polymeric glass formers. This behavior arises from the formation of inter- and intrachain dipole–dipole crosslinks, which causes more dense molecular packing and cohesion.

**References** 

1. Andrew Clark, Yajnaseni Biswas, Morgan E. Taylor, Ayşe Asatekin, Matthew J. Panzer, Christoph Schick, Peggy Cebe. Glass forming ability of polyzwitterions. Macromolecules 2021;54(21):10126–10134. “

### 5.3. Nanocalorimetry Study of 20 nm Thin-Film Ge2Sb2Te5 at 1,000,000 K/s

ZhaoJieAllenLeslieUniversity of Illinois at Urbana-Champaign
jiezhao5@illinois.edu

**Abstract:** Nanosecond switching speeds in phase-change memory (PCM) devices make its material-level kinetic investigation inaccessible for most calorimetric methods. In this work, nanocalorimetry enters this “no-man’s land” with a scanning rate up to 1,000,000 K/s and a sample size (10–40 nm thick) typical of thin-film PCM devices. Negligible thermal lag and no delamination are confirmed in this work. Thin-film GST crystallization is confirmed as a single-step Arrhenius process up to 290 °C dominated by the growth of interfacial nuclei with an activation energy of 2.36 eV. Based on numerical simulation, crystallization growth velocity (CGV) is found to be consistent with that of actual PCM cells.

### 5.4. Crystallization of Polyamide 66/Polyamide 6 Blends

ZhangXiaoshi^1^*RhoadesAlicyn^1^BuzinkaiJohn^2^1Penn State Behrend2INVISTA Nylon Polymer*Correspondence: amh234@psu.edu

**Abstract:** Polyamide 66 (PA66) blends offer a good balance between material properties and cost. A good understanding of the processing of PA66 and its blends at conditions including fast cooling or high undercooling is attracting industry interest. In order to study crystallization under such conditions, flash scanning chip calorimetry (FSC) has been utilized to study PA66 and its blends with polycaprolactam (PA6). Subsequent experiments using WAXS, Micro-IR, and POM have been carried out on samples mounted on FSC chips. Isothermal analysis of the crystallization rate indicates that crystallization in blends becomes slower with a higher blend content. Interestingly, bimodal temperature-dependent kinetics, observed in the PA66 homopolymer, gradually merges into a unimodal crystallization kinetics profile as the PA66 blend fraction decreases. Besides the kinetics, heat of fusion of PA66, PA6, and their blends show a local minimum, which is reminiscent of the previous finding in long-spaced polyacetals. The mechanism that leads to the changes in isothermal crystallization kinetics will be discussed.

### 5.5. Nanocalorimetry for Transmission Electron Microscope

YiFeng^1^*ArlingtonShane^1^McGiesonIsak^2^LaVanDavid^1^1National Institute of Standards and Technology2Oregon State University*Correspondence: feng.yi@nist.gov

**Abstract:** Nanocalorimetry is a chip-based thermal analysis technique capable of measuring phase transformations and chemical reactions at very high heating and cooling rates. Here, a nanocalorimeter was designed to fit inside Titan transmission electron microscope (TEM) holders with an electrical connection design to enable in situ TEM observation during high-rate thermal measurements even with very narrow TEM pole pieces. A 10 nm alumina membrane was realized as the TEM observation window using a lithography step to provide high-resolution images and enhanced diffraction data. For certain samples (such as a 25 nm Au film), the alumina may be bypassed entirely such that the sample can be imaged directly. The elimination of the silicon nitride under the TEM observation region reduced the background signal by eliminating the contributions from the amorphous silicon nitride. Results from preliminary measurements demonstrate the versatile nanocalorimeter for in situ TEM observation. Combining the nanocalorimeter with a TEM equipped with subframing and a high-frame-rate direct electron detection camera will further extend the measurement capability to couple direct microstructural evaluation with thermal analysis.

### 5.6. Flash DSC Investigation of Polyamorphism and Nanocrystallization

PerepezkoJohn*KimWanDuanTianruiCaoChengrongUniversity of Wisconsin-Madison*Correspondence: perepezk@engr.wisc.edu

**Abstract:** The polyamorphism exhibited by D-Mannitol between the normal melt quenched glass (GN) and the amorphous Phase X (GX) induced by annealing has been examined in a detailed series of differential scanning calorimetry measurements covering a wide range of scanning rates. In the glass transition of the GN, TgN develops an increasing behavior upon annealing, but in the glass transition of GX, TgX changes little during annealing, implying that (GX) is a kinetically more stable glass. A series of interrupted thermal cycles have allowed for the identification of a liquid–liquid transition between the supercooled liquid of GN, SCL-1, and that for GX, SCL-2. Under the action of an applied stress, GX can be induced to transform irreversibly to the higher-density GN. The precise annealing conditions that can be reached by flash DSC enabled the construction of the temperature–time–transformation (TTT) plot of D-mannitol for the transition between GN/(SCL1) and G X/(SCL2), as well as the transition between amorphous and crystalline phases revealing thermally activated behavior. In order to analyze the kinetics further, the first order liquid–liquid transition (LLT) in a single-component liquid D-mannitol has been examined in detail by high rate of flash differential scanning calorimetry measurements (FDSC). By controlling the annealing temperature, the Phase X formation from supercooled liquid is distinguished by either a nucleation growth or a spinodal decomposition type of LLT. In the measured time–temperature–transformation (TTT) curve, the portion covering the nucleation growth type of LLT can be well fitted with a classical nucleation theory analysis. During the crystallization of Fe-based metallic glasses, the precipitation of the BCC-Fe nanocrystals smaller than the exchange length (30-40 nm) has been reported as an effective strategy to achieve a reduced coercivity and an increased saturation magnetization. In Fe-B alloys without the addition of nonmagnetic additives, it has been shown that small nanocrystals well below the exchange length can be obtained after rapid heating up to 500 K/s. In order to determine whether heating rates above 500 K/s can yield a further refinement in nanocrystal size, the influence of the heating rate (0.17 K/s to 5000 K/s) on the crystallization behavior of an amorphous Fe85B15 alloy and the nanocrystal size reduction were investigated using Flash DSC and TEM. Above the heating rate of 500 K/s, the number density and diameters of BCC-Fe nanocrystals become saturated due to diffusion field impingement that enriches the amorphous matrix with a B concentration that leads to the precipitation of Fe3B nanocrystals that deteriorate the magnetic properties.

## 6. Session: Kinetics

### 6.1. Selection of the Suitable Kinetic Analysis Method for Polymer Decomposition, Curing and Crystallization by Kinetics Neo

MoukhinaElenaNETZSCH Geraetebau GmbH
elena.moukhina@netzsch.com

**Abstract:** There are several methods of kinetic analysis in NETZSCH Kinetics Neo software, and therefore very often the question comes as to what method is more suitable for one or another polymer process. The first group of processes in polymers is the decomposition. Usually, the decomposition rate can be accurately measured by thermogravimetry. The easiest method for kinetic analysis is the model-free method, such as the Friedman, Vyazovkin or numerical methods. The Ozawa or Kissinger methods were created for single-step reactions and therefore produce good fit only for the first reaction step in multi-step reactions. Another possible method is the model-based method, but here again many possibilities of different reaction types exist. The decomposition of polymers is usually an n-th order reaction. For many solid–solid decomposition reactions, the decomposition is a phase-boundary reaction with the phase border moving in the material. The phase-boundary reaction is described by an n-th order reaction with non-integer n. The typical models are R2 with n = 1/2 and R3 with n = 2/3. The mixture of independently decomposed materials can be described as the independent reaction steps. The second group of polymer processes is the curing and cross-linking reactions. The main measurement technique in use is DSC, which can detect the exothermal curing reactions. Alternatively, the rheological data can be used for kinetic analysis because rheology provides good information about the strong increase in viscosity during curing. The curing reactions are typically autocatalytic, and therefore can be described by the model-based method, where reaction rate is proportional to the product concentration. According to our experience, reactions with autocatalysis such as C1, Cn, Cmn, Bna or Kamal–Sourour are the best reaction types for the kinetic analysis of curing processes. However, if the same reaction can be described well by different autocatalytic equations with different numbers of kinetic parameters, then the user should select that which has the lowest number of adjusting parameters. Some curing reactions take place in the glass transition range where the reaction rate is diffusion-controlled. The vitrification and diffusion control depend on the current glass transition temperature, and therefore could not be described by model-free methods. In this case, we usually select the kinetics according to diffusion control with a given dependence of Tg vs. conversion according to di Benedetto equation. If the viscosity data are used for kinetic analysis, then it is necessary to remove the temperature dependence of viscosity for both the reactant and product. Crystallization processes in polymers are kinetic processes too. This exothermal process can be measured by DSC and analyzed by kinetic methods as well. The crystallization just below melting point can be easily described by the Arrhenius approach. The results of such analysis are applicable only in the small temperature range near the melting point. If the crystallization for cooling is to be analyzed, then the influence of diffusion region near glass transition should be taken into account. The classical model is the Nakamura model with Hoffmann–Lauritzen theory and Avrami dependence on conversion. However, the better solution for many polymers is obtained by the Sbirrazzuolli model with Hoffman–Laurithen theory for diffusion and the Sestak–Berggren model for the nucleation term. This work contains many kinetic application examples for all three groups of processes in polymers.

### 6.2. Kinetic Analysis and Simulation of Non-Isothermal Crystallization Rate of Polymers by Kinetics Neo

MoukhinaElenaNETZSCH Geraetebau GmbH
elena.moukhina@netzsch.com

**Abstract:** Crystallization in polymers is the exothermal kinetic process which can be measured by DSC and then analyzed by kinetic methods. Usually, the crystallization rate is measured under isothermal conditions or during cooling. However, for semi crystalline polymers the recrystallization can be observed during heating, between glass transition temperature and melting temperature. The mechanism of polymer crystallization consists of the nucleation and subsequent crystal growth. Under the isothermal conditions, the crystallization mechanism is described by the Avrami nucleation equation, where the nucleation rate is proportional to the non-crystallized volume of material. The isothermal crystallization just below melting point can be easily described by the Arrhenius approach with model-free analysis, or using model-based analysis with Avrami or Sestak–Berggren reaction types. This provides temperature dependence with a pure mathematical negative value of the apparent activation energy, because nucleation rate increases with lower temperature because of supercoiling. The results of such kinetic analysis are enabled only in the small temperature range near melting point. If the crystallization during cooling is to be analyzed, then the influence of the diffusion region near glass transition should be taken into account. The very fast cooling can be a reason for incomplete crystallization. The kinetic analysis for cooling crystallization is model-based analysis with two parts: nucleation near the melting point and diffusion near the glass transition temperature. The classical Nakamura model with Hoffmann–Lauritzen theory and Avrami dependence on conversion where melting and glass transition are taken into account can sometimes fit the crystallization rate. However, the better solution for many polymers is obtained by the Sbirrazzuolli model with Hoffman–Laurithen theory for temperature dependence and the Sestak–Berggren model for conversion dependence. This model effectively describes the shape and different areas of measured crystallization data during cooling. It can be successfully used for the kinetic analysis of crystallization processes for many polymers. This work contains many examples of kinetic analysis and simulations of crystallization processes in polymers during cooling, isothermal conditions and heating.

### 6.3. Kinetic Modeling of Competitive Epoxy Curing Reactions of Solvay 977-3 Film

SauerbrunnSteve*DeitzelJosephUniversity of Delaware—CCM*Correspondence: sauerbru@udel.edu

**Abstract:** Epoxy curing is often modeled as a single step reaction. Sometimes the curing chemistry is a sequential two-step mechanism. The work reported here is for the curing of Solvay 977-3 film. The best kinetic model was a two-step mechanism where the two reactions are in competition for the unreacted epoxy. The best kinetic model is used to predict the extent of curing for any time–temperature profile. A good kinetic model allows one to simulate the final curing for any changes to the manufacturer’s prescribed curing profile.

### 6.4. Cure Kinetics of an Epoxy Molding Compound for Semiconductor Packaging

TaoRan^1^PhansalkarSukrut Prashant^2^ForsterAaron^1^HanBongtae^2^1National Institute of Standards and Technology2University of Maryland/College Park*Correspondence: ran.tao@nist.gov

**Abstract:** Epoxy molding compounds (EMC) are highly filled thermosetting epoxy polymers used for semiconductor encapsulation since the 1970s. Today’s development trend in semiconductor manufacturing is towards higher-density, thinner and smaller devices. This has increased the demand for EMC to support up-to-date packaging technologies. Accurate modeling of the thermo-mechanical behavior of EMC during curing is critical for reliability prediction and curing process optimization for semiconductor packages. During processing, the epoxy cures, whereby the low-viscosity monomers and oligomers undergo chemical reactions that increase the molecular weight and form a cross-linked network structure. In the final product, the material reaches a fully cured state. Since today’s packaging occurs at the wafer level rather than the device level, issues related to residual stress and warpage due to cure (chemical) and thermal shrinkage, as well as additional stresses developed during thermal cycling became more pressing challenges for the industry. Successful predictive modeling of the process-dependent viscoelastic properties of EMC is imperative for accurate reliability prediction (e.g., stress and warpage) in semiconductor packaging research and development. Understanding the cure kinetics is the first step toward modeling the cure-dependent thermo-mechanical properties of EMC. In this work, we perform both non-isothermal and isothermal experiments to study the cure kinetics of a commercial EMC using differential scanning calorimetry. The reaction kinetic parameters, including the reaction rate expressions, Arrhenius pre-exponential factor, and activation energy, are reported. In addition, the relationship between the glass transition temperature (Tg) and the conversion is obtained by performing isothermal experiments on partially cured samples.

### 6.5. How Advanced Kinetics, Sample Control Thermal Analysis (SCTA) and Heat Balance Can Help Reduce Curing Time and Prevent Runaway

RoduitBertrand^1^*FollyPatrickSarbachAlexandre1AKTS SA;2armasuisse*Correspondence: b.roduit@akts.com

**Abstract:** For highly exothermic reactions, processing, design, quality control and operational applications require an understanding of thermal hazards and an ability to predict safety limits and the decomposition process in extended temperature ranges. Several methods have been presented for the prediction of the reaction progress of exothermic reactions under heat accumulation conditions [1–3]. However, because decomposition reactions usually have a multistep nature, the accurate determination of the kinetic characteristics is difficult, which, in turn, strongly influences the ability to correctly describe the progress of the reaction. The use of simplified and conservative kinetic models for the assessment of runaway reactions leads to economic drawbacks, since they may result in unnecessarily large safety margins. In the present study, we show that applying the results obtained by means of (i) differential scanning calorimetry (DSC) and (ii) advanced kinetic analysis, the prediction of the thermal behavior of the material in the sample controlled thermal analysis (SCTA) mode is possible. The application of the kinetic parameters in SCTA allows the prediction of the reaction course for the temperature ramp, which is required to obtain the chosen, set by user, constant reaction rate. However, knowledge of the kinetics is only one prerequisite during the scale-up of DSC results. The second important issue is the precise heat balance of the system. When increasing the sample mass, the heat cannot be fully exchanged with an environment (as during DSC), which may result in (i) a temperature increase in the samples and, finally, (ii) thermal runaway. This study presents the advantages of combining numerical simulations with advanced kinetic analysis, SCTA and precise heat balance to investigate situations, which are not experimentally accessible due to timing or safety reasons. The advantage of the proposed approach is illustrated by a study of the resin thermosetting: the time of curing can be significantly shortened and the probability of thermal runaway considerably reduced.

**References** 

1. Roduit B.; Hartmann M.; Folly P.; Sarbach A.; Brodard P.; Baltensperger R.; Thermal decomposition of AIBN, Part B: Simulation of SADT value based on DSC results and large scale tests according to conventional and new kinetic merging approach. Thermochim. Acta, 2015, 621, 6.
2. Roduit B.; Hartmann M.; Folly P.; Sarbach A.; Dejeaifve A.; Dobson R.; Kurko K.; Kinetic analysis of solids of the quasi-autocatalytic decomposition type: SADT determination of low-temperature polymorph of AIBN. Thermochim. Acta, 2018, 665, 119.
3. AKTS-Thermokinetics Software (Thermal Aging and Thermal Safety versions), AKTS SA, TechnoPôle 1, Sierre, Switzerland, https://www.akts.com

## 7. Session: Pharmaceuticals and Biopolymers

### 7.1. Sustainable Smart Silk Biomaterial with Tunable Water-Responsiveness Properties

PrintonKyleNewkirkChadMillerWalterHuXiaoRowan University
hu@rowan.edu

**Abstract:** The preliminary focus of this research is to produce and characterize an active silk material that can autonomously fold from a 2D film to a final 3D geometry while also abiding by green chemistry and sustainable engineering principles. The silk biomaterial was dissolved in a formic acid–calcium chloride solution and was left to degrade for time periods ranging from 30 min to 6 days to determine its effects on silks protein structure. The regenerated film is a water responsive (WR) material that can expand or contract in response to changes in relative humidity (RH). Degradation time was investigated as a mechanism for tuning the ratio of β-sheet and random coil regions to control the volumetric strain and amount of water absorbed to fine tune actuation. Fourier-transform infrared spectroscopy (FTIR) analysis confirmed that the frequency of β-sheets (hydrophobic domains) increased by nearly 10% as the degradation period increased. This structural change was also noticed with thermal methods such as thermogravimetric analysis (TGA) and differential scanning calorimetry (DSC). These results were also confirmed with a contact angle analysis and a water absorption study that both determined that longer degraded films are more hydrophobic and less absorbent. A bilayer design, with the 2 h degradation as the active material and the 6-day degradation as the passive material, was determined to generate the greatest actuation angle when compared to single-layer films.

### 7.2. Advantages of Sample Observation Calorimetry for Polymers

FieldingMeaghanC-Therm TechnologiesCorrespondence: meaghan.fielding@ctherm.com

**Abstract:** Calorimetry is a trusted method in thermal analysis that is used to determine thermal events, specific heat, and mass loss. With applications in pharmaceuticals, polymers, and many other areas, DSC and STA are common lab instruments. Recent innovations in calorimetry by Rigaku have resulted in the development of sample observation calorimetry. Sample observation technology allows for the accurate determination of heat events with visual confirmation. Changes in shape and color can easily be observed. This is invaluable when trying to differentiate endothermic heat events, such as a melt vs. a decomposition, or exothermic heat events, such as a crystal-crystal phase change vs. a decomposition. This recent innovation in calorimetry has significant benefits for the polymers/plastic industry. Novel materials typically present a challenge to analyze, as there is no way to visually confirm events such as melt, crystallization, etc. These problems are solved with sample observation calorimetry, as any changes to the sample can be viewed in real time. With the turn away from single-use plastics, we are seeing a rise in novel materials which are developed as an alternative. One example of this is PHBH, a biopolymer. Biopolymers have been replacing single-use plastics for applications such as straws. When PHBH is tested on the Rigaku STA8122, there is a small change in baseline at around 150C, which would typically be attributed to noise. However, when using sample observation, it is easy to confirm that this is in fact the melting point. In the past, it has been impossible to visually confirm changes in a sample when measuring with DSC and STA. Now, changes such as colour, shape, size, and phase change can easily be confirmed. This eliminates the ambiguity from data analysis and ensures correct characterization, resulting in less time wasted searching the literature and more confidence in the data.

### 7.3. Application of Advanced Kinetics in Everyday Life: A Novel Approach to Determining the Shelf Life and Remaining Shelf Life of Perishable Goods

RoduitBertrand^1^*LuyetCharly^1^FollyPatrick^2^SarbachAlexandre^2^BaltenspergerRichard^3^1AKTS SA2armasuisse3University of Applied Sciences of Western Switzerland*Correspondence: b.roduit@akts.com

**Abstract:** The problem of assessing the current extent of deterioration of perishable materials and the negative consequences on the possible variation of their properties is of great importance. The evaluation of kinetic parameters from sparse data developed by AKTS [1] offers the solution to this problem. The approach is based on four steps:

(1) Accelerated thermal aging: At least 20 experimental data points collected at different temperatures are required.
(2) Screening of kinetic models: Performing the fitting procedure including one and two-step equations using an unlimited number of models.
(3) Ranking of kinetic models based on statistical analyses: The best/simplest model is identified based on the highest Akaike (AIC) and Bayesian (BIC) statistical scores.
(4) Predict long-term stability and confidence intervals: Predictive bands (99% CI) are obtained by statistical analysis (bootstrap).

After determining the best kinetic models, it is possible to create the time–temperature–transformation (TTT) diagram, which presents the mutual dependence of time, temperature and transformation progress (considered as the reaction extent). This allows the determination of the extent of degradation and excludes the use of materials such as pharmaceuticals or polymers when their degradation exceeds the specification limit. The kinetic approach makes it possible to determine the degradation progression of materials under all environmental conditions and to continuously assess their remaining life. These applications are essential, especially considering the restrictions required for materials decomposing at low temperatures. The proposed solution allows for optimizing storage/shipping and significantly increasing health safety and waste. The kinetic approach is universal and can be used for all kinds of products to determine their shelf life [2–4].

**References** 

1. AKTS-Thermokinetics Software (Thermal Aging version), AKTS SA, TechnoPôle 1, Sierre, Switzerland, https://www.akts.com
2. Clénet D.; Accurate prediction of vaccine stability under real storage conditions and during temperature excursions. Eur. J. Pharm. Biopharm. 2018, 125, 76.
3. Roduit B.; Luyet C.; Hartmann M.; Folly P.; Sarbach A.; Dejeaifve A.; Dobson R.; Schroeter N.; Vorlet O.; Dabros M. and Baltensperger R.; Continuous monitoring of shelf lives of materials by application of data loggers with implemented kinetic parameters, Molecules, 2019, 24, 2217.
4. Evers A.; Clénet D. and Pfeiffer-Marek S.; Long-term stability prediction for developability assessment of biopharmaceutics using advanced kinetic modeling. Pharmaceutics 2022, 14, 375.

## 8. Session: Andrew McGhie Honorary

### 8.1. Thermal Decomposition of Organophosphorus Flame Retardants

HowellBob A.Central Michigan University
bob.a.howell@cmich.edu

**Abstract:** Because of the tendency of traditional organohalogen flame retardants to migrate from a polymer matrix into which they have been incorporated, leading to human exposure and consequent disease, these materials are rapidly being replaced by other less toxic additives. In the main, these are organophosphorus compounds, with those derived from biobased precursors being particularly attractive. The mode of thermal degradation for these compounds is strongly dependent on structure. Simple phosphate esters (high level of oxygenation at phosphorus) usually undergo elimination of phosphorus acids at relatively low temperatures (200–2500 °C). These acids may promote cationic crosslinking in the polymer matrix and consequent char formation at the surface of the degrading polymer. This char layer acts as an insulation barrier to inhibit heat feedback from the combustion zone and reduce the rate of polymer pyrolysis to generate volatile fuel fragments. Phosphonates and phosphonites (low level of oxygenation at phosphorus) may undergo initial degradation via a similar process but at a higher temperature (300–3500 °C). Subsequent decomposition may lead to the generation of the volatile PO radical which escapes to the gas phase and effectively scavenges combustion propagating radicals. DOPO (9,10-dihydro-9-oxa-10-phosphaphenanthrene-10-oxide) and derivatives are well-known for gas-phase flame retardant activity. Under thermal stress, they efficiently extrude PO radical and form a stable dibenzofuran structure.

### 8.2. Determination of the Cross-Link Density of Ancient Ambers

McKennaGregory^1^KongDejie^2^MengYan^3^1North Carolina State University2Texas Tech University3Beijing University of Chemical Technology
gbmckenna@ncsu.edu

**Abstract:** While it is known that amber results from the free radical polymerization of plant resins, it is also widely believed in the paleontology community that amber is a cross-linked polymer, based primarily on the observation that it is difficult to dissolve in a single solvent. However, presently, there have been no reports of the cross-link density or molecular weight between cross-links of these fascinating materials. In addition, there are data suggesting that the degree of cross-linking may be less than thought. In the present study, we have used dynamic mechanical measurements on a series of ambers with ages from 20 million years to approximately 100 million years to determine the plateau modulus of the materials. By assuming that the ambers form tetrafunctional networks, we estimate the cross-link density for the measured ambers to range from approximately 1800 g/mol to 3000 g/mol, consistent with the cross-link density not being extremely high. It is also of interest that that the age in the amber correlated better with the cross-link density than it does with the glass transition temperature. We will discuss the ambers and their thermomechanical properties as used to determine the cross-link density. Suggested network structures will also be shown.
**Acknowledgments:** The authors thank the National Science Foundation under grants CBET-1603943 and DMR-1610495, and the J.R. Bradford Endowment at Texas Tech University, each for partial support of this project. The authors also would like to give special thanks to Professor Xiaoyu Li at Beijing University of Chemical Technology for his support of this work.”

### 8.3. Developments in Titania Filled Nanocomposite Hybrid Materials

MatisonsJanisIntertape Polymer Group
jgmatisons@gmail.com

**Abstract:** The ideal aim of combining inorganic and organic materials is to achieve an optimal combination of the properties inherent to each material, which can then enhance the value of the hybrid nanomaterial in the end application. Inorganic/organic hybrids have the potential for many useful applications. Some nanohybrid materials under development include high-refractive index lenses, graded index optics, optical filters, sensors, as well as passive and nonlinear active waveguides. Previous work has involved the synthesis of titania nanoclusters for use as precursors in the formation nano-composite network via polymerization with methacrylic acid. Such Ti nanoclusters are incorporated into nanocomposites by using polymerizable ligands on Ti filler particles. By incorporating such Ti nanoclusters into methyl methacrylates, the polymeric properties can be improved (for instance the glassy-rubbery transition temperature can be raised, and the onset of polymer degradation can be delayed). Several analytical techniques were used to determine the structure and material properties of the nanocluster-polymer hybrid. These techniques included FTIR, NMR (C13 and H1), TGA, DSC, ellipsometry, AFM, TEM and SANS.

### 8.4. A Half Century of Research and Educational Outreach at the Laboratory for Research on the Structure of Matter, LRSM

McGhieAndrewLRSM, University of PA
mcghie@lrsm.upenn.edu

**Abstract:** This talk will cover the period, 1970–2022, that I spent at the LRSM, an NSF Materials Research Science and Engineering Center, MRSEC, and will cover some major breakthroughs in research in which I have collaborated. These include work on organic materials purification and single crystal growth and their optical and electronic properties, metallic organic charge transfer compounds, e.g., TTF-TCNQ, polydiacetylenes, conducting polymers, e.g., polyacetylene and polyaniline, which resulted in Nobel prizes for Heeger, MacDiarmid, and Shirakawa in 2000, graphite intercalation compounds, and fullerenes and fullerites, e.g., C60, and C70, all of which have benefitted from the application of thermal analysis techniques. In addition, I developed educational outreach programs in our MRSEC for high school and college students, teachers, and the general public. I was also involved in starting the Partnership on Research and Education in Materials, PREM, a program between a minority college and a MRSEC. Again, thermal analysis techniques were introduced into these programs. During this period, I also joined the TAFDV and NATAS in the late 1970s and worked in the thermal analysis field both by organizing local, TAFDV, national, NATAS, and international, ICTAC, meetings and serving in a variety of committee and executive positions.

### 8.5. Polymer Infiltrated Nanoporous Metal Scaffolds: Effect of Confinement on Thermal Properties

CompostoRussellUniversity of Pennsylvania*Correspondence: composto@seas.upenn.edu

**Abstract:** Adding nanoparticles (NPs) to polymers is now a common route to change and tune properties such as the rolling resistance in automotive tires. However, the aggregation of discrete NPs limits the volume fraction of additives that can be used. To achieve high loadings (ca. 50 vol. %), we infiltrate polymer directly into a stable nanoporous metal (NPM) scaffold. Polystyrene (PS) and poly(2-vinylpyridine) (P2VP) films are infiltrated into the ca. 43 nm-diameter NPG pores via capillary forces during thermal annealing above the polymer glass transition temperature (Tg). The infiltration process is characterized in situ using spectroscopic ellipsometry. PS and P2VP, which have different affinities for the metal scaffold, exhibit slower segmental dynamics compared to their bulk counterparts when confined within the nanopores, as measured through Tg. The more attractive P2VP shows a 20 °C increase in Tg relative to its bulk, while PS only shows a 6 °C increase at a comparable molecular weight. The infiltrated polymer, in turn, stabilizes the gold nanopores against temporal coarsening. The broad tunability of these polymer/metal hybrids represents a unique template for designing functional network composite structures with applications ranging from flexible electronics to fuel cell membranes.

### 8.6. Working with Cellulose to Fabricate Advanced Materials

CruzDavid Salas-de laRutgers University*Correspondence: david.salas@camden.rutgers.edu

**Abstract:** Transforming natural resources from their native state to a more usable form is non-trivial. Natural polymers tend to form extensive networks of hydrogen bonds, which can organize in multiple ways and stabilize a rich variety of crystalline or amorphous phases. Consequently, developing a quantitative methodology and new chemical routes that identify critical interactions and predict how chemical substitutions will impact the properties of the material is valuable and transformative both for improving existing materials and for discovering new ones. This seminar aims to present several research investigations in understanding the structure, morphology, and physicochemical properties of biomacromolecules composites. The first study looks into understanding the morphological changes of cellulose upon dissolution in ionic liquids. The second study will look at a blend of cellulose and silk. When blended, these structures interact hydrophobically and electrostatically; the resulting matrices formed by the blend will exhibit new and useful properties. Changes in polymer ratio, solvent temperature and type, and coagulation agent type play significant roles in the material solubility, thus affecting the final material properties of the blend. Finally, the third study looks into how ionic liquids can be utilized as a solvent, not only to dissolve cellulose but also to functionalize it. A variety of 1,2,3-triazolium-functionalized cellulose derivatives are synthesized while their alkyne chain lengths and counter anions varied to investigate their structure/property relationships. A unique set of characterization tests were used for these investigations, including Fourier transform infrared spectroscopy (FTIR), thermal gravimetric analysis (TGA), differential scanning calorimetry (DSC), nuclear magnetic resonance (NMR), X-ray scattering and ionic conductivity.

## 9. Session: Polymers and Vitrimers

### 9.1. Preparation of Ultra-Stable Polymer Glasses by Vacuum Pyrolysis Deposition

BannaAmer El^1^*McKennaGregory^2^1Texas Tech University2North Carolina State University
a.banna@ttu.edu

**Abstract:** Ultra-stable amorphous fluoropolymer glasses, showing fictive temperature (Tf) reductions of up to 62 K below the nominal glass transition temperature (Tg) exhibited by the rejuvenated material (Tg = 348 K), were created using vacuum pyrolysis deposition (VPD). Glass films with a thickness ranging between 200 and 1150 nm were grown by deposition onto substrates having temperatures ranging from 0.75 Tg to 0.95 Tg, where Tg is that of the virgin material, as reported by the manufacturer, with units in K (381 K). Glassy films were characterized using rapid-scanning chip calorimetry (Flash DSC), Fourier-transform infrared spectroscopy (FTIR) and intrinsic viscometry measurements. Large enthalpy overshoots, the signature of highly aged or ultra-stable glasses, were observed upon heating at 600 K/s. A Tf reduction of this magnitude has not been previously reported and exceeds Tf reductions witnessed in 20–200 million-year-old ambers as well as that reported for a similar deposition of an amorphous Teflon material. This approach suggests a very powerful tool for the exploration of material dynamics deep in the glassy state, in particular for a broad range of temperatures where Tf < T < Tg.

### 9.2. Molecular Vibrations and the Solid-State Heat Capacities of Polyzwitterion and Salt Complexes

ThomasJohnCebePeggyBiswasYajnaseniAsatekinAyseTufts University
lang.wei@dupont.com

**Abstract:** Polyzwitterions (PZIs) have been reported to have strategic properties such as ionic conduction, thermal stability, and mechanical stability, which make them strong candidates for use as solid polymer electrolytes [1]. In this study we investigate the thermal and structural properties of the solid state for three PZI/salt complexes, poly(sulfobetaine methacrylate), PSBMA, poly(sulfobetaine acrylate), PSBA, and poly(ethyl sulfobetaine methacrylate), PESBMA, with and without added salt. PZI/salt complexes were created by dissolving LiCl into the PZI at varying weight percentages of 1–20 %wt LiCl and then solution casting to create thin films. Using temperature-modulated differential scanning calorimetry, the solid-state heat capacity and change in heat capacity at the glass transition (ΔCp) were measured as a function of salt content. Using Raman spectroscopy and Fourier transform infrared spectroscopy, changes in the molecular vibration confirm the association of LiCl with the zwitterionic moiety. The changes in molecular vibrations contribute to a drastic shift in the glass transition temperature, the ΔCp, and the shape of the glass transition relaxation region.

**References** 

1. Cardoso, J., et al., Synthesis and characterization of zwitterionic polymers with a flexible lateral chain. The Journal of Physical Chemistry C, 2010. 114(33): p. 14261-14268.”

### 9.3. Influence of Moisture on Glass Transition Temperature and Mechanical Properties of Epoxy Resin Using DMA with Controlled Relative Humidity

SauerbrunnSteve*DoshiSagarDeitzelJosephGillespieJohn W.Jr.SchneiderAlexUniversity of Delaware—CCM*Correspondence: sauerbru@udel.edu

**Abstract:** Epoxy resins are widely used in composites due to their mechanical properties, thermal stability and relative ease of processing and have various applications from automotive to aerospace. They are often subjected to a wide range of environmental conditions from extreme temperatures to high relative humidity. Epoxies are known to susceptible to moisture, leading to the degradation of properties due to moisture ingress. The effect of moisture on epoxy is not only the deterioration of mechanical properties, but also the suppression of the glass transition temperature. Typically, ASTM D7028 [1] is the standard used for characterizing dry and wet Tg by DMA. However, the standard over-simplifies the challenges involved in wet Tg measurement and does not consider the heat and mass transfer mechanisms. Slower rates cannot be used for measuring the wet Tg due to the drying of specimens, and faster rates could lead to a temperature gradient in the specimen. Researchers have also developed a procedure which involved the development of a diffusion model for the material along with temperature correction data using a thermocouple in the center of the specimen [2]. In this research, we use a novel piece of equipment, a dynamic mechanical analyzer (Netzsch DMA Eplexor) fitted with a chamber to maintain relative humidity, to study the influence of moisture on glass transition temperature and mechanical properties of epoxy resin SC-15. Specimens will be run at ASTM-recommended heating rates (5 °C/min) as well a slow heating rate of 0.5–1 °C/minute. Preliminary results for the tests conducted for specimens with and without controlling relative humidity at the slow heating rate indicate a shift in the onset of Tg and show evidence of a loss of moisture in the Tan Delta curves. About a 20 °C difference in the Tg (by Tan Delta) for specimens is observed when the relative humidity is maintained while running the DMA.

**References** 

1. ASTM D 7028. “Standard Test Method for Glass Transition Temperature (DMA Tg) of Polymer Matrix Composites by Dynamic Mechanical Analysis (DMA),” ASTM International
2. Lee, C. William et al. “Characterization of Wet-Glass-Transition Temperatures for High Temperature Polymers.” SAMPE Conference (2014).

### 9.4. Developing Sustainable, Reprocessable Polymer Networks and Network Composites: Important Roles of Thermal Analysis via Dynamic Mechanical Analysis and Rheometry

TorkelsonJohnNorthwestern University*Correspondence: j-torkelson@northwestern.edu

**Abstract:** One of the holy grails of sustainable polymers is the achievement of recyclability of polymer networks by “melt-state” reprocessing with full recovery of cross-link density and associated properties after recycling. At present, almost no polymer thermosets or thermoset composites are recycled for high-value applications, making the 9% of all thermoplastics that are recycled worldwide seem like a major success by comparison. The production of conventional crosslinked polymer networks and their composites, i.e., thermosets and thermoset composites, was estimated at 40 billion kg of polymer in 2020. Unfortunately, thermosets cannot be melt-reprocessed into high-value products because the permanent crosslinks prevent melt flow. Examples include rubber tires and polyurethane (PU) foam, with major economic and sustainability losses as a result. Here, we report on research employing simple one-step or two-step reactions to produce networks with dynamic covalent crosslinks that are robust at use conditions but allow for melt-state reprocessing via dissociative or associative exchange reactions at a high temperature. We developed three approaches that allow for melt-state reprocessing of addition-type polymer networks and network composites, including those synthesized directly from monomers containing carbon–carbon double bonds and those synthesized from combined polymers and monomers with both containing carbon–carbon double bonds. These approaches allow for full crosslink density recovery after multiple reprocessing steps. Using four dynamic chemistries, we also made reprocessable PU and PU-like networks, from polyhydroxyurethane (PHU) and polythiourethane (PTU), with full recovery of crosslink density. An “Achilles’ heel” is associated with dynamic covalent networks, i.e., they are subject to creep at elevated temperature. We addressed this limitation in several ways. First, we replaced a fraction of dynamic covalent crosslinks with permanent crosslinks. Second, we used dynamic chemistry with a high activation energy, allowing for reprocessability at high temperature but with the dynamic chemistry essentially fully arrested well above room temperature, e.g., 70–100 °C. Dynamic mechanical analysis (DMA) and rheometry are the key analytical tools to characterize full or partial success in achieving recovery of cross-link density and associated properties in recycled networks as well as suppressed elevated temperature creep behavior. In particular, DMA and rheometry allow for exacting the characterization of key property recovery and performance of recycled networks with a very small error as a function of temperature, as will be demonstrated with results from recent studies [1–3].

**References** 

1. Li LQ, et al., Vitrimers designed both to strongly suppress creep and to recover original cross-link density after reprocessing: Quantitative Theory and Experiments. Macromolecules 2018;51;5537-5546.
2. Hu SM, et al., Biobased Reprocessable Polyhydroxyurethane Networks: Full Recovery of Crosslink Density with Three Concurrent Dynamic Chemistries. ACS Sustain. Chem. Eng. 2018;7;10025-10034.
3. Bin Rusayyis MA, Torkelson JM. Reprocessable and recyclable chain-growth polymer networks based on dynamic urea bonds. ACS Macro Lett. 2022;11;568-574.

### 9.5. The Problem of Temperature Error: Revisiting the Anomalous Structural Recovery in a Polymer Glass

JinShuang*McKennaGregoryNorth Carolina State University*Correspondence: sjin8@ncsu.edu

**Abstract:** In the isothermal structural recovery of polymer glass, the departure from equilibrium is generally viewed as a smooth function of time, as shown in the dilatometric studies by Kovacs [1]. However, recent studies by Boucher et al. [2] and Cangialosi et al. [3] reported two-mechanism structural recovery in enthalpy at aging temperatures ranging from 6 to 17 K for polystyrene, drawing considerable interest. In an attempt to replicate the results, Koh and Simon [4] conducted one-year isothermal aging experiments on polystyrene of similar molecular weight at 15 K and did not observe the intermediate plateau in the two-mechanism structural recovery. There remains important work to determine what could cause non-smooth structural recovery. In this study, we test the hypothesis that poor temperature control that is typical of a vacuum oven could lead to the anomalous results. We use the Tool–Narayanaswamy–Moynihan (TNM) model to calculate the structural recovery of polystyrene undergoing such thermal histories that were affected by the vacuum oven temperature variation and find that the reported two-mechanism structural (enthalpy) recovery can be reproduced when such temperature variability was considered.

**References** 

1. Kovacs AJ. In Fortschritte Der Hochpolymeren-Forschung. Springer, Berlin, Heidelberg. 1964; 3: pp. 394-507.
2. Boucher VM, Cangialosi D, Alegria A, Colmenero J. Macromolecules. 2011;44:8333.
3. Cangialosi D, Boucher VM, Alegria A, Colmenero J. Phys Rev Lett. 2013;111:095701.
4. Koh YP, Simon SL. Macromolecules. 2013;46:5815.

## 10. Session: Energetic Materials

### 10.1. Studies of New Catalysts Derived from Ferrocene for the Highly Efficient Combustion of Solid Rocket Motor Propellant Using Thermal Methods

ValdebenitoCristianGaeteJoséOsorioClaudioAbarcaGabrielMoralesCesarUniversidad Bernardo O’Higgins
camoralv@uc.cl

**Abstract:** Rocket technology research is currently receiving the utmost attention due to its potential applications, mainly in the aerospace equipment industry. In this way, for the first time in human history, a commercially built spacecraft by Space-X operated by a crew of NASA astronauts was launched from American soil to the International Space Station. Furthermore, the launch of this commercial space system designed for humans is an essential step on our path to expand human exploration to the Moon and Mars. A propellant rocket motor is the purest form of an energy conversion device in which matter (solid or liquid) is burned, producing hot gases. Concerning the solid propellant rocket, the thermal decomposition of ammonium perchlorate (AP) has a close relationship with the propellants’ combustion process. There are highly effective burn-rate (BR) catalysts for AP and excellent candidates for application in rocket engines with high thrust and acceleration power in comparison with Fe_2_O_3_, a commonly used BR catalyst. Therefore, the impact on the thermal degradation of AP is often used to evaluate the combustion effect of a burning rate catalyst candidate on the combustion behavior of composite solid propellants. This work aims to describe the catalytic effect of different BR catalysts derived from ferrocenes by thermogravimetry and differential scanning calorimetry techniques, which will significantly decrease the decomposition temperature of ammonium perchlorate, resulting in improved performance of the composite solid propellant and increasing the energy release.

### 10.2. Thermal Screening of Reactive Chemical Process from DSC Measurements

WeiLangMortonPaulDupont
lang.wei@dupont.com

**Abstract:** Differential scanning calorimetry (DSC) is useful for the chemical characterization of materials. Additionally, DSC is increasingly used to assess the thermal hazards of potentially energetic compounds or to calculate the viability of chemicals for storage in harsh environments. At DuPont we use DSC to investigate and compare the reaction chemistry/thermal stability of materials. In this presentation, we will use DSC to compare “identical” monomers from three different vendors. The monomer samples were prepared and sealed in capillary glass tubes and a heat–cool–heat mode was run at a constant rate. The DSC results illustrate that the samples are not identical, and we observed different detected onset temperatures of exothermic activity, which were consistent with subsequent accelerated rate calorimeter (ARC) results. Compared with other reaction chemistry tools such as ARC, VSP, and TSU, the DSC measurement has the advantages of low cost, fast results, and convenient operation and maintenance.

### 10.3. Determination of CL-20 Melting and Liquid Thermal Decomposition Kinetics Using Fast Scanning Calorimetry

DentonAric^1^*SimonSindee^1^McKennaGregory^2^1Texas Tech University2North Carolina State University*Correspondence: aric.austin.denton@gmail.com

**Abstract:** Extensive conventional DSC measurements have been published in the literature for CL-20 (hexanitrohexaazaisowurtzitane), but even at the highest heating rates (>100 K min^−1^, 3.3 K s^−1^), CL-20 thermally decomposes before reaching its melting point. Here, we describe a work in which we used rapid chip calorimetry (FlashDSC™) to separate the decomposition temperature from the melting temperature. By heating at rates from 100 K/s to 20,000 K/s, we were able to, for the first time, observe melting in CL-20. By heating at very high rates, we avoided the kinetically limited thermal decomposition behavior before observing a (thermodynamic) melting endotherm in the CL-20 that showed its onset at approximately 280 °C. We continued heating the now liquid CL-20 until the completion of thermal decomposition. Using the known heat of decomposition, we could estimate the mass of CL-20 on the Flash DSC chip, which was then used to estimate the heat of melting. In addition to the melting behavior, the decomposition kinetics and activation energies for the liquid CL-20 were determined. The range of heating rate data for the solid phase decomposition kinetics was extended by 3 decades from previous works reported in the literature.
**Acknowledgments:** The authors thank the National Armament Consortium (NAC) and Defense Ordnance, Technology consortium (DOTC) for partial support for this project under agreement DOTC-16-01-INIT0177. Support was also provided by BAE Systems, Ordnance Systems Inc. under award number HID-031521-01-MJP. We also thank the Office of Research and Innovation at Texas Tech University, the J.R. Bradford Endowment, the Whitacre College of Engineering, and the Department of Chemical Engineering each for partial support of this work.

### 10.4. Explosive Compatibility with Fuels and Firefighting Chemicals

GuillenGingerLawrence Livermore National Laboratory*Correspondence: guillen5@llnl.gov

**Abstract:** The compatibility of explosives is extremely important to avoid any potential sensitization or negative effects to the explosives’ thermal stability. Compatibility in abnormal chemical environments must also be analyzed to avoid increased hazards and establish a safe protocol and a suitable approach. In the event of an accident or fire, explosives stored nearby may be exposed to common chemicals in the vicinity (e.g., gasoline) or chemicals used to mitigate the hazard (e.g., firefighting chemicals). These chemicals and compounds need to be considered to avoid any adverse reactions to the explosive itself, especially at elevated temperatures. In already dangerous conditions, fighting a fire with materials that are proven to be incompatible with certain explosives could lead to increased hazards. In addition to immediate effects or thermal runaway, an incompatibility can alter the explosive’s chemical properties, causing it to not function as originally expected. Poor functionality due to exposure or direct contact with an incompatible material could cause a future hazardous situation as well. Thermal analysis was performed on a wide array of explosives with various materials that could be used as firefighting chemicals and fuels. The objective of this work is to screen firefighting materials and other compounds that could potentially be in direct contact with explosives in fueling or fighting a fire. Differential scanning calorimetry (DSC) compatibility was used to analyze TATB-, LLM-105-, RDX, HMX-, PETN-, and TNT-based formulations in combination with commercial aircraft fuel, civilian and military gasoline/diesel, and firefighting solid, liquid, and gas chemicals. Upon an incompatible result through DSC analysis, additional compatibility testing can be carried out through other thermal analytical techniques such as using thermogravimetric analysis (TGA) or simultaneous thermal analysis–mass spectrometry (SDT-MS) to corroborate these results. Furthermore, we can thermally accelerate the aging of the materials in question through the chemical reactivity test (CRT) to verify compatibility and obtain an accurate representation of these concerns.

### 10.5. Why Can the Safety Parameters of Energetic Materials (TMRad, SADT) Change during Storage?

RoduitBertrand^1^*FollyPatrick^2^SarbachAlexandre^2^1AKTS SA2armasuisse*Correspondence: b.roduit@akts.com

**Abstract:** This study presents the results of simulations of the influence of the material aging degree (aaging) on its thermal behavior at the milligram (as in DSC) and kilogram scale (SADT) [1–4]. The simulations were performed for the autocatalytic reaction (Prout–Tompkins kinetic model, PT) and first-order reaction (F1). The simulation results show that the influence of aging on the decomposition course is significant for materials decomposing according to the autocatalytic models and negligible for those decomposing according to the first-order kinetics. The autocatalytic character of the reaction can be easily uncovered by a double scan test (DST) [3], which requires only two non-isothermal runs with identical heating rates. The DST allows the comparison of the thermal behavior of samples with slightly different aging values in two subsequent experiments. In addition, we present simulations of the dependence of the thermal properties of materials on aging under real-world climatic conditions [3,4]. The presented results indicate that the aging degree of materials should be considered an essential parameter, next to the commonly used kinetic triplet, when predicting sample properties.

**References** 

1. United Nations, UN Recommendations on the Transport of Dangerous Goods, Manual of Tests and Criteria, 5th ed., United Nations, New York and Geneva, (2009) 297-316.
2. Roduit B.; Hartmann M.; Folly P.; Sarbach A.; Brodard P.; Baltensperger R.; Thermal decomposition of AIBN, Part B: Simulation of SADT value based on DSC results and large scale tests according to conventional and new kinetic merging approach. Thermochim. Acta, 2015, 621, 6.
3. Roduit B.; Hartmann M.; Folly P.; Sarbach A.; Parameters influencing the correct thermal safety. Evaluations of autocatalytic reactions. Chem. Eng. Trans., 2013, 31, 907.
4. AKTS-Thermokinetics Software (Thermal Aging and Thermal Safety versions), AKTS SA, TechnoPôle 1, Sierre, Switzerland, https://www.akts.com

## 11. Session: Crystalline Polymers

### 11.1. Silk-Based Magnetic Nanofiber Materials with Tunable Structures and Properties to Enhance Biological Responses

HuXiaoRowan University
hu@rowan.edu

**Abstract:** There has been increasing attention paid to protein-based smart materials for use in diverse applications due to their excellent biocompatibility and high flexibility. In this study, flexible silk fibroin protein and biocompatible barium hexaferrite (BaM) nanoparticles were combined and electrospun into nanofibers, and their physical properties could be tuned through the mixing ratios and a water annealing process. Structural analysis indicates that the protein structure of the materials is fully controllable by the annealing process. The mechanical properties of the electrospun composites can be significantly improved by water annealing, while the magnetic properties of barium hexaferrite are maintained in the composite. Notably, in the absence of a magnetic field, cell growth increased slightly with increasing BaM content. The application of an external magnetic field during in vitro cell biocompatibility study of the materials demonstrated significantly larger cell growth. We propose a mechanism to explain the effects of water annealing and magnetic field on the cell growth. This study indicates that these composite electrospun fibers may be widely used in the biomedical field for controllable cell response through applying different external magnetic fields.

### 11.2. Alternative Approaches for Non-Isothermal Crystallization Kinetics of Polymers

SchaweJürgenMettler-Toledo GmbH
juergen.schawe@mt.com

**Abstract:** We present two approaches to describe the non-isothermal crystallization of polymers during cooling from the melt. The first is an empirical model to describe the dependence of the enthalpy of crystallization on the cooling rate, while the second is based on a thermodynamic model of crystallization and describes the dependence of the crystallization temperature on the cooling rate. The empirical model is very sensitive in determining the influence of modifications such as fillers and nucleating agents on the crystallization process. If the sample is measured in a sufficiently large cooling rate range, the critical cooling rate can be determined. This is a sensitive property that can be used to detect variations in the overall crystallization rate. This approach is applied for various nucleating polypropylenes. Further information about the crystallization process can be derived using the thermodynamics-based model. The dependence of the crystallization temperature on the cooling rate is characterized by a kinetic factor κ, which depends on the critical work to form secondary nuclei during the growth process. According to the Hoffman–Lauritzen theory, this parameter is expressed by the surface free energies of the polymer crystals. This approach is applied to several polymers measured by Flash DSC. It is shown that the combination of flash DSC and Eq. (1) allows a fast and sensitive identification of thermodynamic parameters related to primary polymer crystallization.

### 11.3. Thermal and Raman Spectroscopic Analysis of Alpha-Phase Poly(vinylidene fluoride)

JayasekaraAnujaCebePeggyTufts University
anuja.jayasekara@tufts.edu

**Abstract:** Poly(vinylidene fluoride), PVDF, is a semi-crystalline polymer with three common crystalline polymorphs [1]. The most common polymorph is the non-polar alpha-phase which has crystals with a net zero dipole moment due to its TGTG’ chain conformation and chain packing in alpha-unit cell [1]. We investigate the relationship between thermal and spectroscopic properties of PVDF to develop alternative methods for determining the crystal phase and the degree of crystallinity of the different polymorphs. In this work, we concentrate on the alpha-PVDF polymorph. Films were prepared with different crystallinities and examined using Fourier-transform infrared spectroscopy (FTIR), differential scanning calorimetric (DSC), and Raman spectroscopy. FTIR confirms that our samples contain solely alpha-phase PVDF. Crystalline fraction, fc, was controlled by cooling from the melt at various rates. fc was evaluated from fc = Hf/Hf0, where Hf is the DSC measured heat of fusion and Hf0 is the equilibrium heat of fusion of alpha-phase PVDF (Hf0 = 104.5 J/g) [2]. Raman spectroscopic analysis has been used in determining the crystalline fraction of many polymers [3–5] but, until this work, it has not been applied to PVDF. Raman peaks corresponding to alpha-phase PVDF crystals were identified by temperature-controlled Raman peak analysis. A comparison between the degree of crystallinity determined by DSC and Raman spectroscopic analysis is presented.

**References** 

1. Lovinger AJ, Poly(Vinylidene Fluoride), in Developments in Crystalline Polymers—1, D.C. Bassett, Editor. 1982; Springer Netherlands: Dordrecht: 195-273.
2. Sajkiewicz P, Steady-state process and time-dependent effects in non-isothermal crystallization of poly (vinylidene fluoride). Polym. 1999; 40(6): 1433-1440.
3. Agarwal UP, RS Reiner, and SA Ralph, Cellulose I crystallinity determination using FT–Raman spectroscopy: univariate and multivariate methods. Cellulose. 2010; 17(4): 721-733.
4. Kotula AP, CR Snyder, and KB Migler, Determining conformational order and crystallinity in polycaprolactone via Raman spectroscopy. Polym. 2017; 117: 1-10.
5. Nielsen AS, D Batchelder, and R Pyrz, Estimation of crystallinity of isotactic polypropylene using Raman spectroscopy. Polym. 2002; 43(9): 2671-2676.”

### 11.4. Competition between Heterogeneous Nucleation and Flow-Induced Crystallization of Polyamide 66 and Its Carbon Nanotube Composites

GohnAnne^1^*ZhangXiaoshi^1^McHaleAlexander^1^AndroschRené^2^RhoadesAlicyn^1^1Penn State Behrend2Martin-Luther-University Halle-Wittenberg*Correspondence: amh234@psu.edu

**Abstract:** Heterogeneous nucleation and flow-induced entropy reduction are the two well-known factors that accelerate polymer crystallization. However, the interplay of nucleation and flow-induced acceleration is still poorly understood. This work investigates the nucleating effect of carbon nanotubes (CNT) on both the quiescent and flow-induced crystallization kinetics of polyamide 66 (PA 66). The quiescent crystallization study indicates that CNT acts as a powerful nucleant, as suggested by the fact that the critical cooling rate to bypass crystallization and create the amorphous glassy state changes from 1000 K/s in PA 66 neat resin to a rate faster than 4000 K/s in the PA 66 nanocomposites. The flow-induced crystallization study indicates that PA 66 onset crystallization time and morphology depend on the shear work introduced by rotational rheometry. A combined acceleration effect from CNT nucleants and FIC persists when the CNT loading is below the saturation limit. However, if CNT loading meets the saturation limit, specific shear work shows no impact on the crystallization time, providing evidence that the role of the FIC acceleration effect no longer exists when heterogeneous nucleation acceleration dominates the crystallization of PA 66.

### 11.5. Effect of Grafting Density on the Crystallization Behavior of PHEMA-g-PEO Bottlebrushes

WilkJeffrey^1^*FurnerCarl^1^KellyMichael^2^ZhaoBin^2^LiChristopher^1^1Department of Material Science and Engineering, Drexel University, Philadelphia PA 191042Department of Chemistry, University of Tennessee, Knoxville TN 37996*Correspondence: jtw79@drexel.edu

**Abstract:** In recent years, there has been a renewed interest in utilizing brush polymers for targeted applications. One system of interest is bottlebrush polymers, which comprise hyperbranched polymer chains grafted to a semi-flexible backbone. The chemical tethering of the side chain to the backbone imparts interesting physical constraints on the polymer chain, inducing the chain to stretch orthogonally while increasing the rigidity of the backbone. This results in unique properties of these systems, such as reduced side-chain entanglement, the formation of large domain sizes and rapid phase separation [1–3]. The crystallization behavior of tethered chains is significantly different from its homopolymer counterpart. A significant factor in bottlebrush crystallization behavior is the influence of side-chain packing, or grafting density. Grafting density dictates the equilibrium state of the brush and provides additional chain density towards a growing crystal. In the dilute crystallization state, molecular bottlebrushes have been shown to form hollow sphere structures upon crystallization, with radial size dictated through asymmetric packing and controlled through grafting density [4]. Characterization of the influence of grafting density in the bulk state will provide information on the physical state of the side chain and promote the development of targeted applications. To investigate this phenomenon, a series of poly(2-hydroxyethylmethacrylate)-g-poly(ethylene oxide) (PHEMA-*g*-PEO) bottlebrush polymers were synthesized via a copper catalyzed azide-alkyne cycloaddition reaction. The grafting density was finely controlled by varying the feed molar ratio of PHEMA backbone monomer units to PEO, yielding a series of bottlebrush samples with grafting densities ranging from 19 to 94%. The bottlebrushes were then subjected to a series of differential scanning calorimetry measurements (DSC). Non-isothermal DSC and Hoffman–Lauritzen analysis showed a peak crystallinity and equilibrium melting temperature for the 73% grafted sample, suggesting an optimal architecture for promoting crystallization. Polarized light microscopy revealed that nucleation is significantly increased for intermediate grafting densities, promoting the rapid development of crystalline microstructure. Additionally, isothermal DSC measurements yielded characteristic Avrami exponents ranging from 1.6 to 2.4, suggesting that the geometry of the initial nucleus is primarily disc-like. Additionally, a thermal fractionalization protocol was developed to investigate the retention of nuclei beyond the nominal melting point of the bottlebrush. The higher grafting densities exhibit a higher isotropic melting point than their sparsely grafted counterparts, suggesting that the stretched geometry of the brush helps to preserve residual orientation developed in the crystalline state, while crystallization at lower grafting densities is inhibited through the defect contribution of the backbone. This work provides a systematic insight into the crystallization behavior of grafted chains and can provide insights into the physical state of highly grafted chains as well as providing information for the development and processing of bottlebrush polymers.

**References** 

1. Verduzco R, Li X, Pesek SL, Stein GE. Structure, function, self-assembly, and applications of bottlebrush copolymers. Chem Soc Rev. 2015;44(8):2405-2420.
2. Nikovia C, Theodoridis L, Alexandris S et al. Macromolecular Brushes by Combination of Ring-Opening and Ring-Opening Metathesis Polymerization. Synthesis, Self-Assembly, Thermodynamics, and Dynamics. Macromolecules. 2018;51(21):8940-8955.
3. Hu M, Xia Y, McKenna GB, Kornfield JA, Grubbs RH. Linear rheological response of a series of densely branched brush polymers. Macromolecules. 2011;44(17):6935-6943.
4. Qi H, Liu X, Henn DM et al. Breaking translational symmetry via polymer chain overcrowding in molecular bottlebrush crystallization. Nat Commun. 2020;11(1):1-9.

### 11.6. Thermal Analysis as an Efficient Tool to Monitor the Process–Structure–Property Relationships in Crystalline Polymer Processing: 1. Fiber Formation

JaffeMichaelNew Jersey Innovation Institute*Correspondence: jaffe@njit.edu

**Abstract:** Over the past several decades, much of what had been a thriving US fiber production industry has moved to countries with developing economies and large populations. This has led to a diminution of fiber R&D in US (and western European) universities, corporations and government laboratories. While some work persists, mainly in the areas of biomedically relevant fiber constructs and nanofiber production, little R&D remains in the understanding and optimization of the large-scale production of fibers for textile, industrial or medical applications.

## 12. Session: 3D Printing/Additive Manufacturing

### 12.1. Thermal Behavior of Fe-6.5Si Alloy Powder for AM-SLM Processing as Assessed by Differential Scanning Calorimetry (DSC)

PetrovicDarja Steiner^1^SeverTina^1^DonikCrtomir^1^PaulinIrena^1^GodecMatjaz^1^PetrunMartin^2^1Institute of Metals and Technology, Ljubljana, Slovenia2University of Maribor, Faculty of Electrical Engineering and Computer Science, Slovenia
darja.steiner@imt.si

**Abstract:** Soft magnetic intermetallic alloys with up to 6.5% Si possess excellent soft magnetic properties: high permeability, low coercivity, near-zero magnetostriction, and high saturation induction, making them ideal for use in electromagnetic devices. However, as the Si content is increased, the material becomes extremely brittle, and it is very difficult to produce thin sheets by conventional rolling. Additive manufacturing (AM) has been identified as a technique that enables the processing of bulk near-net-shape geometries of intermetallic soft magnetic alloys at optimal compositions. The main advantages of AM technologies compared to conventional synthesis and shaping processes of functional magnetic materials lie in their ability to produce complex and/or customized parts with a short lead time. A successful AM processing and implementation of novel custom-tailored magnetic materials into electromagnetic applications would enable a next-generation design of electric devices and would result in drastic energy, material, and cost savings. Lab-scale processing that enables AM-SLM of small powder volumes for materials development is of special importance because materials may be studied under very well-controllable conditions. In this study, a high-alloy, Fe-Si spherical powder (45 µm in diameter) was investigated. Calorimetric techniques, such as differential scanning calorimetry (DSC) and differential thermal analysis (DTA), are often applied for the determination of reaction kinetics upon heating and cooling. The solidification that occurs during cooling is of particular interest for technical alloys. Differential scanning calorimetry, thermodynamic calculations, and metallographic analyses were used for a determination of the solidification sequence in the Fe-6.5Si alloy. The dynamic measurements involved two consecutive cycles at the same selected ramp rate, i.e., 10 K/min. The linear temperature program for heating and cooling was as follows: 25 °C–1550 °C–350 °C–1550 °C–25 °C. There was no isothermal annealing performed at maximum temperature. Subsequently, the loss of the alloying element Si was investigated by classical analytical method, i.e., gravimetry. It is well known that chemical composition crucially determines the magnetic properties of non-oriented electrical steel sheets. Due to the technical importance of Fe-Si alloys and an increasing need to improve their magnetic properties, the aim of the present study was to determine the solidification path in the selected alloy powder, and to determine the loss of silicon during processing. Based on the results of differential scanning calorimetry, thermodynamic calculations, and metallographic analyses, the melting as well as the solidification behavior of the Fe-6.5Si powder are evaluated and compared by the conventional non-oriented electrical steel [1].

**References** 

1. Klancnik G., Medved J., Nagode A., Novak G., Steiner Petrovic D. Influence of Mn on the solidification of Fe-Si-Al alloy for non-oriented electrical steel, JTAC 2014;116:295–302. “

### 12.2. Kinetics of Crystallization for Polyamide 12 during Selective Laser Sintering for 3D Printing

MoukhinaElenaRudolphNatalieSchmoelzerStefanNETZSCH Geraetebau GmbH
elena.moukhina@netzsch.com

**Abstract:** Selective laser sintering (SLS), also called power bed fusion (PBF), is the layer-by-layer construction technology of 3D objects by a laser beam which passes over selected places on a powder layer. The powder of the working material (usually a polymer) is melted in these places by the laser and then crystallized. Then, it stays solid and retains the shape of the molted geometry. Currently, PA12 is widely used, but some materials with improved properties are still under construction. The process window and crystallization behavior of the new materials are very important to know in order to find optimal temperatures for 3D printing. These temperatures are one of the main parameters of sintering process influencing the speed of sintering and quality of end product. The manual optimization of this process for new materials could take a very long time. It can be done much faster using NETZSCH Kinetics Neo software for the kinetic modelling of crystallization rate based on DSC data, and then the simulation of process for different temperature profiles. Firstly, the experimental DSC measurements should be carried out, then kinetics analysis is performed for these data and the kinetic model is created. Finally, the kinetic model is used for the simulation at different temperatures in order to find the optimal one. The simulations for standard printing and high-speed printing are presented, and their influence on the properties of the final product is discussed.

### 12.3. Tracking the Properties that Govern Structural Stability of Printed Thermosets during Curing via Rheo-Raman Microscopy

RombergStian*KotulaAnthonySeppalaJonathanNational Institute of Standards and Technology*Correspondence: stian.romberg@nist.gov

**Abstract:** With their ability to be extruded at room temperature and excellent compatibility with fillers, thermoset composite resins show the potential to expand the material properties and scale offered by direct ink write (DIW) additive manufacturing. However, the factors governing structural stability during printing and curing are a key challenge to successful thermoset DIW. Recent work linked the storage modulus and yield stress to the height at which printed thermoset structures collapse under their own weight, but did not investigate structural stability during post-curing at elevated temperatures. At elevated temperatures, rheological properties of thermosets are governed by a competition between the temperature dependence of the resin and the progression of the curing reaction, impeding characterization with conventional techniques. The rheo-Raman microscope, which can simultaneously measure rheological properties and probe reaction progress via the Raman spectrum, can provide critical insights into the dependence of these properties on temperature and degree of reaction. This work uses the rheo-Raman microscope to track rheological properties that have been shown to affect stability at a range of isothermal cure temperatures. High temperatures can cause the storage modulus and yield stress to drop before these properties increase due to the crosslinking reaction. Lower cure temperatures can mitigate this initial drop in properties but increase the time needed to reach the final extent of reaction. By combining the results from the temperature tests on the rheo-Raman microscope with previously developed mechanical models, recipes can be designed to maintain the structural stability of printed parts during curing while minimizing the time required to reach full curing. This study demonstrates a new way to evaluate and design thermoset composites for post-cured DIW and provides an understanding of the limits of these materials for thermoset additive manufacturing applications.

### 12.4. 3D Printing via Direct Ink Writing (DIW) of Self-Healing Elastomers

RibeiroErick L.^1^*BucknerEmily^1^CaldonaEugene^1^AdvinculaRigoberto^2^1The University of Tennessee, Knoxville2Case Western Reserve University*Correspondence: eribeiro@utk.edu

**Abstract:** Autonomous self-healing elastomers have attracted much attention owing to their capacity to enhance the lifespan, stability, and safety of products. The ability to utilize such materials to manufacture complex structures via three-dimensional (3D) printing techniques is highly desired and has the potential to impact several fields, ranging from medicine to aerospace and the electronics industry. Nonetheless, the complexity arising from the self-healing elastomers’ intrinsic polymeric structure and rheological properties has restricted their 3D printing applications to manufacturing only at lab-scale operations [1]. Herein, we present our findings on the addition of poly(BCOE), a well-known autonomous self-healing polymer (SHP), to a commercially available silicone rubber (Dow DOWSIL^®^ 795). More specifically, we investigated the dispersion of poly(BCOE) in the commercial adhesive matrix in different mass ratios (0, 10, 30, and 50 wt%), and their corresponding effects during the production of tensile bars via direct ink writing (DIW). FTIR spectra of SHP samples confirm the presence of urethane group hydrogen bonds, which are associated with its autonomous self-healing properties. DSC reveals that the crystallization temperatures of Dow795 and 10 wt%-SHP/Dow795 are both −45 °C. In addition to that, TGA curves demonstrate the thermal degradation temperature range, where an increase in the mass ratio of the SHP with respect to the adhesive matrix resulted in a lower degradation temperature. Such findings confirm the successful dispersion of poly(BCOE) in the Dow795 matrix. Incomplete degradation indicates that constituent materials within Dow795 undergo thermal degradation at higher temperatures. These findings indicate the successful dispersion and the resulting interaction between poly(BCOE) and the adhesive matrix, with can be tailored to further tune the properties of the Dow795.

**References** 

1. Ritzen, L., Montano V., Garcia S.J. 3D Printing of a Self-Healing Thermoplastic Polyurethane through FDM: From Polymer Slab to Mechanical Assessment. Polymers 2021; doi:10.3390/polym13020305

### 12.5. Rheological Study on Direct Ink Writing of Bisphenol A-Based Epoxy and Its Composites to Optimize Stability, Printability, and Mechanical Properties

SmithZane*AdvinculaRigobertoUniversity of Tennessee, Department of Chemical & Biomolecular Engineering*Correspondence: zsmith31@vols.utk.edu

**Abstract:** Direct ink writing (DIW), an extrusion-based additive manufacturing (AM) technique, utilizes a viscous and thixotropic ink that, once printed, is chemically cured to obtain the final AM part. To perform DIW with various thermoset materials without crosslink assisted setups, materials need rheological modifiers to make a thixotropic ink for it to withstand its shape during printing. Typically, rheological modifiers such as fumed silica nanoparticles (SNP), clay nanoplatelets, carbon nanotubes (CNT), and graphene are used to create the desired percolation network. Numerous studies have characterized the effects of these rheological additives in epoxy resin to optimize the printing conditions; however, little is known on how these additives completely interact with neat epoxy resin as well as epoxy composites [1–3]. For this work, a rheological study on various nano/micro additives and modifiers for a commercial grade bisphenol A-based (BPA) epoxy resin is performed. Important properties were achieved based on developing the structure–composition–property correlation and concentration dependence of SNP, clay nanoplatelets, CNT, and milled/chopped CF. Spectroscopic and microscopic characterization methods (SEM, TEM, Raman, etc.) were also employed.

**References** 

1. Hmeidat, N. S., Pack, R. C., Talley, S. J., Moore, R. B., and Compton, B. G. (2020). Mechanical anisotropy in polymer composites produced by material extrusion additive manufacturing. Addit. Manuf., 34. doi:10.1016/j.addma.2020.101385
2. Kasraie, M., and Pour Shahid Saeed Abadi, P. (2021). Additive manufacturing of conductive and high-strength epoxy-nanoclay-carbon nanotube composites. Addit. Manuf., 46. doi:10.1016/j.addma.2021.102098
3. Romberg, S. K., Islam, M. A., Hershey, C. J., DeVinney, M., Duty, C. E., Kunc, V., and Compton, B. G. (2021). Linking thermoset ink rheology to the stability of 3D-printed structures. Addit. Manuf., 37. doi:10.1016/j.addma.2020.101621

### 12.6. Optimization Study of 3D-Printed Polyetheretherketone and Its Composites for Biocompatible Applications

DabkowskiDavid*SmithZaneRibeiroErick L.CaldonaEugeneAdvinculaRigobertoUniversity of Tennessee, Department of Chemical & Biomolecular Engineering*Correspondence: ddabkows@vols.utk.edu

**Abstract:** Polyetheretherketone (PEEK) is a high-performance, semi-crystalline polymer known for its chemical resistance, mechanical integrity, dielectric performance, and ability to perform under elevated temperatures [1]. Currently, PEEK is approved by the US Food and Drug Administration (FDA) for surgical implant applications [2], and is commonly employed for many useful biomedical applications including spine treatment, orthopedic tools/products, and dental implants [3]. The 3D printability of pristine PEEK filament has been previously demonstrated for optimized mechanical performance [4]. In our current study, we demonstrate the 3D printing of pristine PEEK (i.e ThermaX™ PEEK) and its composites (i.e., Solvay Ketaspire PEEK+CF10 and CarbonX PEEK+CF20) via fused deposition modeling (FDM). We focus primarily on optimizing the printability of carbon fiber (CF)-reinforced PEEK composites and investigate their biocompatibility for potential biomedical applications. Thermomechanical, topological, dielectric, thermal, mechanical, and antimicrobial tests are used to characterize our printed PEEK materials.

**References** 

1. De Leon, Al Chrisopher C., da Silva, Italo G. M., Pangilinan, Katrina, Chen, Qiyi, Caldona, Eugene B., and Advincula, Rigoberto. High performance polymers for oil and gas applications. United States: N. p., 2021. Web. doi:10.1016/j.reactfunctpolym.2021.104878.
2. Liu, J., & Goode, J. (2018, September 17). Recognized consensus standards. accessdata.fda.gov. Retrieved June 1, 2022, from https://www.accessdata.fda.gov/scripts/cdrh/cfdocs/cfstandards/detail.cfm?standard__identification_no=37390
3. Sunpreet Singh, Chander Prakash, Seeram Ramakrishna, 3D printing of polyether-ether-ketone for biomedical applications, European Polymer Journal, Volume 114, 2019, Pages 234-248, ISSN 0014-3057,
4. Carlo S. Emolaga, Shaun Angelo C. Arañez, Persia Ada N. de Yro, Jocelyn P. Reyes, Brigida A. Visaya, Blessie A. Basilia, and Rigoberto C. Advincula, “Surface Design of 3D-printed PEEK by Controlling Slicing Parameters,” International Journal of Mechanical Engineering and Robotics Research, Vol. 11, No. 3, pp. 181-186, March 2022. DOI: 10.18178/ijmerr.11.3.181-186

### 12.7. Poly(N-vinyl carbazole)-Graphene Oxide Nanocomposites for Dispersion and Thermal Stability in Polymer Blends with Applications in Additive Manufacturing

BucknerEmily*EdaugalJustinRibeiroErick L.AdvinculaRigobertoUniversity of Tennessee at Knoxville*Correspondence: ebuckne6@vols.utk.edu

**Abstract:** Graphene, the world’s strongest and thinnest material, has two main limitations: its solubility in aqueous media and its scalability in industrial applications and manufacturing. A well-researched work-around to this issue is the use of graphene’s oxidized derivative, graphene oxide (GO), as a soluble and more feasible additive in composites [1]. The introduction of GO in composites has the potential to improve mechanical, thermal, electrical, optical, and antimicrobial properties with a range of applications in construction, electronics, sensors, energy storage, the biomedical field, etc. The present study investigates effective methods for the uniform dispersion of GO in various polymer matrices. Initially, poly(N-vinyl carbazole)-graphene oxide (PVK-GO) nanocomposites were prepared by solution blending methods to stabilize GO through embedment in the PVK matrix. The immobilization of GO enables the production of various GO composites where the PVK-GO nanocomposite may be blended with other polymer matrices. The 3D printing of GO composites enhances the mechanical strength and thermal stability typically lacking in additively manufactured parts. Thus, the PVK-GO nanocomposites were incorporated into commercial resin and silicone rubber for stereolithography (SLA) and direct ink write printing, respectively.

**References** 

1. Santos, C.M.; Tria, M.R.; Vergara, R.V.; Ahmed, F.; Advincula, R.C.; Rodrigues, D.F. Antimicrobial Graphene Polymer (PVK-GO) Nanocomposite Films. Chem. Commun. 2011, 47, 8892-94.

### 12.8. Thermomechanical Optimization Study on the Extruding Temperature Effects of FDM 3D Printed PPSU and PPS

GoliasCullen^1^*SmithZane^2^ViersDrew^2^RibeiroErick L.^2^AdvinculaRigoberto^2^1JIAM2University of Tennessee, Department of Chemical & Biomolecular Engineering;*Correspondence: cgolias@vols.utk.edu

**Abstract:** Fused deposition modeling (FDM) is an extrusion-based technique of additive manufacturing (AM) that consists of partially bonded layers of a polymer and voids between these bonds. The quality of bonding and size of voids are dependent on the processing settings implemented during the printing process. This technique can be used on a wide variety of polymers and compounds, including high-performance polymers (HPP) with desirable properties for many engineering applications. Polyphenylsulfone (PPSU) and polyphenylene sulfide (PPS) are HPPs that have desirable properties for a wide range of potential applications. Specifically, PPSU is a biocompatible amorphous structure with high impact resistance, as well as hydrolysis while maintaining a high stability under elevated pressures and temperatures [1]. PPSU has potential applications in the aviation, plumbing, electrical, and medical fields. Similarly, PPS is a semi-crystalline thermoplastic that has high chemical resistance properties and exceptional tribological properties. PPS also has many potential applications in the medical, electrical, automotive, and plumbing fields [2]. This work presents our findings on the optimal extrusion conditions (e.g temperature, software-process parameters) for FDM-printed PPSU and PPS filaments via TGA, DSC, rheology, SEM, tensile and flexural characterization, which provide the basis for a comprehensive structure–property relationship analysis relating the printing filaments to their resulting printed-parts, also presented herein. In the future, a continuation of the characterization testing of PPSU and PPS and introducing other forms of testing such as Izod testing and fracture testing will be performed.

**References** 

1. POLYPHENYLSULFONE (PPSU, PPSF). PPSU. (n.d.). Retrieved June 1, 2022, from https://polymerdatabase.com/Polymer%20Brands/PPSU.html
2. Polyphenylene sulfide (PPS) - A robust polymer with multiple applications: Polymers from Poly Fluoro Ltd. Poly Fluoro Ltd. (2019, November 13). Retrieved June 1, 2022, from https://polyfluoroltd.com/blog/polyphenylene-sulfide-pps-a-robust-polymer-with-multiple-applications/#:~:text=%20%20%201%20It%20finds%20uses%20in,engineering%2C%20PPS%20is%20used%20for%20various...%20More%20

### 12.9. 3D-Printed Poly(vinylidene fluoride)-co-hexafluoropropylene (PVDF-HFP)/Silica Superhydrophobic Membranes for Application in Oil/Water Separation Processes

KilpatrickLucas^1^,^2^,^3^*CaldonaEugene^1^,^2^RibeiroaErick L.^2^RongLihan^5^ChengXiang^5^AdvinculaRigoberto^1^,^2^,^4^,^5^1Department of Chemical and Biomolecular Engineering, Tickle College of Engineering, University of Tennessee, Knoxville, USA2Institute for Advanced Materials and Manufacturing, University of Tennessee, Knoxville, USA3Tennessee Technological University, Cookeville, USA4Oak Ridge National Laboratory, TN, USA5Case Western Reserve University, Cleveland, OH, USA*Correspondence: lkilpat3@vols.utk.edu

**Abstract:** Superhydrophobic materials repel water by combining the effects of low surface energy and a hierarchical surface roughness. Membranes constructed from such materials find application in high-efficiency oil and water separation, where oil flows freely through the membrane while water is selectively rejected. However, traditional manufacturing techniques and materials limit the size and geometry of such membrane constructions. The present work investigates the design possibilities for superhydrophobic materials through the manufacturing and characterization of 3D printed superhydrophobic membranes. A superhydrophobic polymer composite “ink” was created by dissolving poly(vinylidene fluoride)-*co*-hexafluoropropylene (PVDF-HFP) in dimethylformamide (DMF) and subsequently adding different ratios of hydrophobic fumed silica (Cab-O-Sil TS-530). Each ink solution was 3D printed via direct ink writing (DIW) with a HyRel Engine SR into regular grid lattice structures with 0.4 mm line width and gap spacing. The mechanical properties and hydrophobicity of the printed mesh membranes were determined. Our findings indicate that increasing loadings of silica enhance the hydrophobic effect, with 30% w/w of silica relative to PVDF-HFP content being the maximum practicable concentration. This material achieved a water contact angle of 161±1° while also exhibiting good tensile and flexural strength. The separation efficiency of the 30% silica membranes was assessed with mixtures of water and various common oils. Additionally, the membrane surface micro and mesostructure was imaged by scanning electron microscopy (SEM). Measurements of surface energy, water contact angle, and sliding angle were performed using a KINO SL250 contact angle meter. Emulsion breaking and anti-icing capabilities were also evaluated

### 12.10. 3D Printing Adhesive and Elastomeric Materials: Characterization and Optimization

ElaheeG M Fazley^1^*RongLihan^1^ChengXiang^1^GeJin^1^BretingChase^1^YangMatthew^1^XuMingwei^1^Bonilla-CruzJose^2^CenicerosTania Ernestina Lara^2^SmithZane^3^CaldonaEugeneRibeiroErick L.AdvinculaRigoberto^1^1Case Western Reserve University2Advanced Functional Materials & Nanotechnology Group, Centro de Investigación en Materials Avanzados S. C. (CIMAV-Unidad Monterrey)3University of Tennessee, Department of Chemical & Biomolecular Engineering*Correspondence: gxe43@case.edu

**Abstract:** The development and optimization of efficient, low-cost, yet environmentally friendly manufacturing methodologies have become a central theme in our technology-driven societies. In recent years, direct ink writing (DIW) has emerged as a promising manufacturing methodology owing to its high-quality patterning capabilities. Specifically, DIW enables multi-material depositions via a precise computer-aided spatial-temporally controlled process, facilitating the fabrication of well-defined geometrical structures based on a broad range of materials. Such precise patterning ability, however, relies on a meticulous tuning in the rheological properties, as well as the composition of the ink, which can lead to a laborious and costly process. In this study, we present a comprehensive characterization of well-known commercially available adhesives and elastomeric materials as inks for DIW printing. More specifically, chemical compositional and rheological characterizations were conducted for several different commercial adhesives and elastomers, which were subsequently evaluated in terms of DIW printing quality, as well as mechanical, electrical, and thermal properties of their corresponding 3D- printed structure. To that end, herein we demonstrate the feasibility of the application of commercially available elastomers as an alternative to complex and costly DIW ink compositions. Additionally, such findings set the ground for the design of a myriad of ink formulations which could be achieved via the introduction of additives, or modifications to the matrix.

### 12.11. 3D Printing of Graphene Oxide Nanocomposites for Biomedical Devices via Digital Light Processing

EdaugalJustin*BucknerEmilyThe University of Tennessee, Knoxville*Correspondence: jedaugal@vols.utk.edu

**Abstract:** Bacterial infections are one of the major reasons of biomaterial implant inefficiency and failure. Traditional implants are prone to bacterial growth, which can lead to infection and pathogen-based diseases. Furthermore, implant failures may require antibiotic treatment and, in some cases, full surgical removal, which may be timely, costly, and can increase the health risks of patients. Recently, various biocompatible materials have been researched to replace commonly used metals in traditional implants. However, these materials can be prone to a weak mechanical strength and thermal instability. Interestingly, 3D printing, coupled with the use of carbon-based nanomaterials, hold much potential to revolutionizing the production of biomedical implants and devices, allowing for greater design specificity and rapid prototyping. This project evaluates the addition of a nanofiller, graphene oxide (GO) to a commercially available photopolymer acrylate resin to improve the thermomechanical strength and antimicrobial activity of a 3D printed nanocomposite for biomedical applications. Additionally, this project evaluates GO combined with poly(*N*-vinylcarbazole) (PVK-GO) as a polymer blend to a commercially available photopolymer resin to further improve the dispersion and thermal stability and strength. Minimal amounts of GO/PVK-GO (0.0, 0.05, and 0.1 wt%) were loaded into a standard Elegoo acrylate resin. Samples were 3D printed via digital light processing (DLP) using an Elegoo Mars Pro and annealed at 50°C and 100°C. GO is characterized via Raman spectroscopy and Fourier transform infrared spectroscopy (FTIR) to confirm the oxidation and OH-group content, respectively. The nanocomposites are characterized via tensile strength (UTM) and thermogravimetric analysis (TGA) to measure the tensile strength and thermal degradation, respectively.

### 12.12. Pyrolysis GC/MS: Application in Facile Characterization of 3D Printing Materials

ChengXiang^1^*ElaheeG M Fazley^1^RongLihan^1^GeJin^1^RibeiroErick L.^2^AdvinculaRigoberto^2^1Case Western Reserve University2The University of Tennessee, Knoxville*Correspondence: xxc337@case.edu

**Abstract:** With the largely expanded use of the 3D printing technique in both research and industry, the components and properties of polymeric materials used in the 3D printing industry vary among different production methods and manufacturers. Components and structures are not easy to study and characterize for polymeric materials, especially after different 3D printing processes. Even though methods such as IR and solid-state NMR spectroscopy are accessible, they are either not sufficient to distinguish structures or require sophisticated interpretation methods. Among all applicable characterization methods, Py-GC/MS is feasible and facile to analyze the polymeric structures, despite thermoplastics or thermosets. The method has a simple sample preparation process and fewer prerequisites for the physical properties of starting samples. Herein, the applications of Py-GC/MS in characterizing polymeric 3D printing materials are demonstrated, from the determination of the main component and additive structures to thermal degradation behaviors, and even to matching molecular weight (Mw) and crosslinking status.

### 12.13. 3D Printed PLA: Making a Commonly Brittle Polymer Elastic and Ductile via Annealing

RohanSalvador^1^*CaldonaEugene B.^3^NiuIvy^3^ChengXiang^3^AdvinculaRigoberto C.^2^,^3^1Department of Materials Science and Engineering, The University of Tennessee, Knoxville, TN 37916, USA;2Department of Macromolecular Science and Engineering, Case Western Reserve University, Cleveland, OH 44106, USA;3Department of Chemical and Biomolecular Engineering, The University of Tennessee, Knoxville, TN 37916, USA;*Correspondence: rrohanra@vols.utk.edu

**Abstract:** Poly(lactic acid) (PLA) is an well-known thermoplastic polyester obtained from renewable sources. In addition to that, PLA is the most common material utilized for 3D printing applications owing to its relatively reduced cost, biocompatibility and environmentally friendliness. Semi-crystalline PLA commonly has strain values around 10%, making it brittle yet strong among several 3D printed materials. However, it lacks the elasticity seen in other materials such as polypropylene, another commonly 3D printed material, which can have strain values around 18% [1]. Herein we present our most recent findings on the effect of the variation of processing parameters on the mechanical properties of tensile bars produced via the fused deposition method (FDM). In addition to that, the present study evaluates the role played by selected post-treatments, namely chemical and thermal annealing, on the properties of the PLA-printed parts. Specifically, it was observed that with chemical annealing, the strain for these tensile specimens increased up to a maximum of 200% elongation. This particular group showed a plasticization effect that varied with the amount of infill present in each infill group. These findings set the ground for the designing of 3D PLA-based printed structures with selected mechanical properties obtained by tuning the processing parameters along with post-treatment conditions.

**References** 

1. Bhasney, S.; Kumar, A.; Katiyar, V. Composites Part B Engineering 2019, 107717.

### 12.14. High Performance Polymers and Additives: From 3D to 4D Printing and Biobased Nanofillers

AdvinculaRigobertoCase Western Reserve University*Correspondence: rca41@case.edu

**Abstract:** Additive manufacturing or 3D printing can be used to create prototypes and devices from polymeric materials which have appended the design functionality for new materials including uses in biomedical devices enabling their rapid development. While 3D printed polymers can be further classified into thermoplastics, thermosets, and elastomers based on their thermo-mechanical properties, new opportunities for multi-materials and composites are possible. Four-dimensional printing allows the design of new materials and applications based on integrating the chemistry of conversion with the printing mode and final stimuli-responsive properties on the printed part. In this talk, we will demonstrate the 3D and 4D fabrication of high-performance polymers and multi-materials including epoxy and benzoxazine thermosets, biobased nanocomposites, and thermoset elastomers with concept objects and elastomeric properties unlocked with stimuli-response. Other works based on the use of SLA, SLS, and FDM 3D printing towards high-strength and nanocomposite materials will be discussed. The importance of thermal, thermo-mechanical, and spectroscopic analysis will be emphasized.

### 12.15. Additively Manufactured Polyvinylidene Fluoride Materials with Optimized Thermomechanical and Electrical Performance

BurchTabitha D.^1^,^2^,^3^,^4^*CaldonaEugene B.^1^,^3^,^4^BethellMichael C.Jr.^2^,^3^,^4^MoczadloMelanie^2^,^3^,^4^PattenCharles G.^1^,^3^,^4^RibeiroErick L.^1^,^3^,^4^AdvinculaRigoberto C.^1^,^2^,^3^,^4^,^5^1Department of Chemical and Biomolecular Engineering;2Department of Material Science and Engineering;3Institute for Advanced Materials and Manufacturing; The University of Tennessee, Knoxville, Tennessee, 37996.4Center for Nanophase Materials and Sciences; Oak Ridge National Laboratory, Oak Ridge, Tennessee, 37830.5Department of Macromolecular Sciences and Engineering; Case Western Reserve University, Cleveland, Ohio, 44106.*Correspondence: tburch5@vols.utk.edu

**Abstract:** Fused deposition modeling (FDM) is an extrusion-based additive manufacturing technique where polymer filaments are printed layer by layer. The print quality and presence of voids in the printed materials are strongly dependent on their printing parameters and post-processing. Polyvinylidene fluoride (PVDF) is a polymorphic semi-crystalline and semi-fluorinated polymeric material used in a wide variety of beneficial applications (i.e., medical, flexible electronic devices, nonvolatile memory, memristive devices, and artificial neutral synapses [1,2]) due to its chemical inertness, thermal stability, low surface energy, nonstick, and piezoelectric properties. Herein, we demonstrate the 3D printability of pristine and additive-containing PVDF filaments via FDM and optimize their printing parameters and post-processing. Spectroscopy and microscopy are used to characterize the structure and morphology of the printed PVDF materials, and thermal, mechanical, dielectric, dynamic mechanical, and contact angle tests are performed to investigate their thermomechanical, electrical, and surface properties, while coming up with a meaningful structure-property relationship analysis. Future work involves testing the compressibility and flexibility of the printed PVDFs as well as their piezoelectric properties.

**References** 

1. Yin, Z., Tian, B., Zhu, Q. and Duan, C., 2022. Characterization and Application of PVDF and Its Copolymer Films Prepared by Spin-Coating and Langmuir–Blodgett Method.
2. Smith, D. W., Iacono, S. T., and Iyer, S. S. (Eds.). (2014). Handbook of fluoropolymer science and technology. John Wiley & Sons.

## 13. Session: Advanced Instrumentation and Tandem Techniques

### 13.1. Microfluidic Platform for Characterizing the Convective Heat Transfer Properties of Suspensions

RosenfeldJoseph^1^LeeDaeyeon^1^AgiralAnil^2^JayneDoug^2^PashkovskiEugene^2^1University of Pennsylvania2The Lubrizol Corporation
jrose92@seas.upenn.edu

**Abstract:** The demand for thermal management fluids of greater efficiency is on the rise due to increased electrification across multiple industries. Conventional thermal management fluids do not possess the heat sink rates to match or exceed the heat source rates in high power applications. Adding various additives is a promising approach toward engineering the properties of thermal management fluids for intended applications. The thermal conductivity of mixtures and suspensions has been extensively studied; however, there are few reports that study their convective heat transfer properties. Additionally, observation of these fluids in situ is rarely undertaken, creating a degree of separation between experimentation and characterization. This work aims to test suspensions for their convective thermal properties using a microfluidic platform comprising a microheater and a PDMS microchannel. An infrared camera is used to measure temperatures of the fluid in situ under laminar flow conditions. The thermal conductivity can be semi-quantitatively assessed by examining the axial temperature profile, while the convective heat transfer coefficient (and by extension the Nusselt number) can be quantitatively assessed via a macroscopic energy balance between Joule heating of the microheater and convective cooling of the fluid. This work aims to aid in the rational design of thermal management fluids with enhanced cooling efficiency.

### 13.2. Introducing the Discovery TMA 450 RH: A Revolutionary New Humidity Controlled Thermomechanical Analyzer

SloughCarltonSaiengaJasonTA Instruments
gslough@tainstruments.com

**Abstract:** Water is everywhere, and humidity can be widely variable depending on location or application. The impact covery TMA 450 RH, which detects changes in dimension, under conditions of both controlled temperature and humidity. The instrument features and benefits will be described in detail with the discussion of numerous real-world applications.

### 13.3. Applications of Coupled Rheology–FT-IR to Polymer Analyses

ReynaudSara*GarciaDanaLavachMarkHenryJamesArkema*Correspondence: sara.reynaud@arkema.com

**Abstract:** The advancement of coupled rheological spectroscopic techniques opens up opportunities to study the in situ structure–property–processing–performance relationships of polymers under dynamic conditions. At Arkema, we explored the use of combined rheo–IR in the attempt to understand the mechanisms behind phenomena such as shear instability, preferential crystallization pathways, structural changes under processing conditions, and more. This presentation summarizes our initial feasibility studies on temperature-dependent structural transitions in styrene–butadiene copolymers and polymethylmethacrylate-polylactic acid (PMMA-PLA) blends. The collection of real time IR spectra allows the identification of chemical changes within the polybutadiene blocks during isothermal rheological experiments. The partial irreversibility of the chemical changes indicates a possible crosslinking of the copolymer when processed at high temperatures. In the case of PMMA-PLA blends, the variation of the IR spectra suggests the formation of a metastable morphology formed at high temperatures, which was difficult to detect by the rheology alone. In a more recent study, we describe the mechanisms of internal lubrication due to the addition of polymer process aids to polyethylene. In particular, we will focus on commercial fluoropolymer-based polymer processing additives, Kynar^®^ PPAs, used to help melt fracture during film extrusion. We show that the addition of a ppm level of Kynar^®^ PPAs into PE drastically improves the quality of extrusion. The lubrication phenomenon is due to the migration of PPA particles to the metal surface of the die, which promotes wall slippage. Although the PPA’s migration mechanism at high shear rates is well understood in the industry, very little is known about the effect of PPAs on the flow behavior of the molten polymer when processed at relatively low shear rates. Our rheo–IR findings indicate a good correlation between the transient viscosity and the evolution of the CH2 band in presence of PPAs. Based on the conformational changes and the increase in PE mobility observed in the IR spectra when shearing PE with Kynar^®^ PPA, we suggest a new internal lubrication mechanism that involves the diffusion of PPA droplets across the polymer matrix instead of migrating to surface. This work explains why a small amount of Kynar^®^ PPA is also beneficial for low-shear rate processing, such as pipe and cable extrusion. Further experiments are underway to investigate the lubrication and migration phenomena in other commercial systems.

### 13.4. On-Line ATR-Fourier Transform Spectroscopy System for Real-Time Quantification of Extrusion-Based Processes

JrLucivan Barros^1^*KleinDana^1^CanevaroloSebastiao^2^MaiaJoao^1^1Case Western Reserve University2Federal University of Sao Carlos*Correspondence: joao.maia@case.edu

**Abstract:** Twin screw extruders (TSEs) are normally used when the application of high stresses and tunable residence times is necessary, which is especially true in polymer blending and compounding and in some reactive extrusion processes. In order to achieve a uniform dispersion of the second phase in the matrix, high levels of dispersive and distributive mixing are needed, leading to high performance properties in blends. However, the effect of process conditions along the extruder barrel can significantly change the dispersion and distributive condition of the melt during its processing, especially when it comes to reactive extrusion. The goal of this work is to develop and validate the on-line Fourier transform infrared spectroscopic set up for the real-time quantification of the melt extrudate during reactive extrusion-based process. The arrangement consists of an infrared spectrophotometer operating in attenuated total reflectance ATR-FT-IR, fitted on-line, along the extruder barrel, thus allowing process kinetics to be studied in near real time. The ATR-FT-IR prototype probe was initially calibrated and validated using off-line measurements. The probe is then interfaced to the extruder through a probe holder unit (PHU), and the on-line ATR-FT-IR measurements are conducted at various locations (L/D) along the length of the extruder. Application examples are shown for immiscible (PP/PA6) and reactive (PP-*g*-AA/PA6) polymer blends, as well as for a TPU polymerization process.

### 13.5. Understanding Adhesive Properties Using Thermal Analysis

StewartCathyIntertape Polymer Group*Correspondence: cstewart@itape.com

**Abstract:** Thermal analysis, in and of itself, is critical in order to understand adhesive properties. However, thermal analysis can also provide clues to an adhesive’s chemistry that feed directly into other analytical techniques. For example, data from TGA decomposition feed into XRF, DRIFTS or heart-cutting GC/MS. The glass transition temperature determined by rheology can be used to predict polymer/resin ratios that are later confirmed by GPC. OIT can be used to determine the correct amount of antioxidant or UV inhibitor in an adhesive and troubleshoot when an adhesive shows poor light or heat stability. Knowing this information, the adhesive can then be pyrolyzed and analyzed by GC/MS to check for the presence and/or changes in AO or UV inhibitors levels. Using DSC to determine Tg and melting points of resins can feed directly back into the rheological behavior of the finished adhesive. Thermal analysis is literally the foundation for adhesive development, raw material qualification, and troubleshooting finished products.

## 14. Session: Alan Riga Honorary

### 14.1. The Chemistry, Structure and Thermal Properties of Starches in Adhesives

MatisonsJanisIntertape Polymer Group
jgmatisons@gmail.com

**Abstract:** Starch is a naturally occurring glucose homo-polysaccharide of nutritional, pharmaceutical, and industrial importance. The complex polymeric structure and poor solubility of native starch in water limits their importance in industry. The structure, reactivity, and functionality of the native starch can be modified by physical, chemical, enzymatic, and biotechnological methods. This talk looks at the latest advancements in characterizing different starches, with particular emphasis on the structure and rheology of pasting starches in relation to adhesive performance. Various physical modification techniques, including the thermal, radio-thermal, freezing and thawing, annealing, and high-pressure methods, and chemical modification techniques, including oxidation, etherification, esterification, cationization, cross-linking, and graft polymerization, can change the surface properties, polarity and linearity of the molecular chains, the degree of substitution, the polymeric, granular, and crystalline structure, amylose to amylopectin ratio, solubility, viscosity, pasting, gelatinization, swelling, water absorption, and emulsifying properties of starch. The structural changes result in the improvement of thermal and freeze–thaw stability, viscosity, solubility, water binding capacity, swelling power, gelling ability, and enzymatic digestibility of starch.

### 14.2. Thermal Decomposition of Organohalogen Compounds in the Presence of Iron Compounds

HowellBob A.Central Michigan University*Correspondence: bob.a.howell@cmich.edu

**Abstract:** Organobromine compounds, particularly brominated aryl ethers, have long been popular flame-retardant additives for polymeric materials. They are inexpensive (diphenyl ether is a byproduct of the Dow process for phenol production) and effective. Poly(vinyl chloride) (PVC) is a widely used halogenated polymer. Antimony oxide is commonly used as an adjuvant for both halogenated polymers and polymer additives. In a degrading polymer or polymer/flame retardant blend, it is converted to volatile antimony trihalide or oxyhalides which may suppress flammability by interfering with gas-phase combustion reactions. However, antimony oxide is both toxic and expensive. More suitable metal compounds to function in this role are being sought. Iron compounds are highly attractive for this purpose. They are readily available, nontoxic and inexpensive. The behavior of several iron compounds in PVC undergoing thermal degradation has been assessed using thermogravimetry (TG) and limiting oxygen index (LOI).

### 14.3. The World of Oxidative Analysis Using Pressure Differential Scanning Calorimetry

AdamsTinaBrowningMeganThe Lubrizol Corporation*Correspondence: tina.adams@lubrizol.com

**Abstract:** The use of pressure differential scanning calorimetry (PDSC) has long been a staple in the repertoire of thermal analysts, especially for the comparison of oxidative stability. How do you know what method to use? What are the implications of your choice? Understanding the myriad of variables possible in developing or choosing an experimental method can propel your project forward to success. Advances in instrumentation and accessories over the years have given the thermal analysis community the best of reliability and accuracy. So, designing the right method falls on the scientist, requiring in-depth knowledge of the sample type being investigated and the goals of the project at hand. Greases, oils, polymers, and bio-based samples all require specific handling and thermo-oxidative profiles for maximum knowledge and benefit to the goals of your team or customer. Method development decisions involve several crossroads that will be discussed in this presentation. Oxidative onset temperature (OOT) can give you knowledge of a new material or unknown sample, while oxidative induction time (OIT) can aid in comparing the relative characteristics within a sample matrix. Next, decisions must be made with respect to sampling and preparation. Today’s selection of sample crucibles is vast, with options for material, shape, and size. The purity of the oxygenated atmosphere can influence the data. The pressure of the gas is used to speed oxidation to a reasonable experimental time period, control flash events with certain chemistries, and ensure full exposure to the oxygen in cases of solid materials, so choosing the appropriate range is key. Then, consider whether your experiment should have flow or be static. Using an industry standard method can put your data on common ground with customers, but in research the approach may need to be customized. Finally, once the thermal curve is gathered, analysis parameters must be consistent. In 1996, Alan Riga and Gerald Patterson organized a symposium in New Orleans sponsored by ASTM Committee E37 on Thermal Methods that included oxidative testing using PDSC [1]. It is time to open these discussions anew!

**References** 

1. A. Riga and G. Patterson, Eds., Oxidative Behavior of Materials by Thermal Analytical Techniques, STP1326-EB, ASTM International, West Conshohocken, PA, 1997

### 14.4. Dielectric Relaxation in Blends of PVDF with Zwitterionic Copolymers

CebePeggy*ClarkAndrewMonteroMiriam SalcedoGovinnaNelakaLounderSamuelAsatekinAyseTufts University*Correspondence: peggy.cebe@tufts.edu

**Abstract:** Prof. Alan Riga has used dielectric relaxation analysis to study pharmaceuticals, biomaterials, and polymers. In this presentation, I will first review the fundamentals of dielectric relaxation, including the use of parallel plate electrodes, and the temperature dependence of polarization. Boyd’s model for complex systems, such as semicrystalline polymers, will be described. Dielectric spectra can be analyzed using several different formalisms, including complex permittivity, complex conductivity, and complex modulus. The choice of formalism depends upon the measured spectral shape, and the presence or absence of conductivity as a major component of the spectrum. The modulus formalism is especially useful in the case of ionic polymers, such as the category of polyzwitterions.

### 14.5. Use of MTDSC in the Detection of Weak Glass Transitions: The Hysteresis Peak

MenczelJoseph^1^JindalAditya^2^MallamaciMichael^2^AbrahamTonson1Thermal Measurements LLC2Parker Hannifin Corporation*Correspondence: jmenczel@yahoo.com

**Abstract:** The miscibility of poly(butylene terephthalate) with PN-250 plasticizer was characterized by modulated temperature DSC (MTDSC) measurements. The glass transition parameters were measured by total heat flow measurements as the midpoint of the heat capacity increase (for the PBT rich areas) and by creating an endothermic hysteresis peak at the glass transition of the plasticizer, because there was no low-temperature baseline due to the low Tg of PN-250. The disadvantage of this method is that it does not allow the determination of the heat capacity jump at the glass transition. Partial miscibility was determined between these two components. It was observed that the crystallinity of PBT in the blends was much higher than in neat PBT because of the higher segmental mobility of the polymer segments in the blends. The hysteresis peak at the PBT glass transition in the blends is much narrower and more intense than for neat PBT, since the mobile amorphous fraction of PBT in the blends is higher. Together with the lower rigid amorphous fraction, this means a sharper boundary between the mobile amorphous fraction and the PBT crystallites.

### 14.6. Alan T. Riga the Scientist, Inventor, Scholar and Teacher

JudovitsLawrenceArkema Inc.*Correspondence: larry.judovits@arkema.com

**Abstract:** It is with great pleasure that I would like to recognize Alan Riga for his lifetime achievement in the field of thermal analysis. Alan is well known to the thermal analysis community, having been a NATAS president in 2000 as well as receiving the NATAS Award for Outstanding Achievement, better known as the Mettler Award, in 1998. Alan has also been very active on Committee E37 on Thermal Measurements since 1984, especially regarding his studies on the oxidative behavior of materials. Alan won the ASTM 2009 Dudley Award. This award is the highest technical award offered by ASTM International. Alan received his bachelor’s degree in professional chemistry, an MS as well as a PhD in physical chemistry in 1967, all from Case Western Reserve University, Cleveland, Ohio. He is a fellow of NATAS, ASTM, and Society of Plastics Engineers (SPE). Alan is also an inventor and developed a drug delivery system using a transdermal patch technology. He has a number of patents, which also include three patents issued for safe emulsion explosives. Alan has taught as an adjunct professor. These universities include Case Western Reserve University, University of Toledo, and Cleveland State University. He has been a visiting professor with the University of Sao Paulo, Brazil. Finally, he and his wife Judy have been married for 60 years, and they have three children and six grandchildren.

## 15. Session: Batteries

### 15.1. Anisotropic Thermal Conductivity Considerations for Application in Battery Thermal Management Systems

HakimianAryaC-Therm Technologies Ltd.*Correspondence: arya.hakimian@ctherm.com

**Abstract:** The thermal management of battery systems, especially those in electric vehicles (EVs), has garnered a lot of attention in recent years. A key challenge in advancing battery technologies is tied to temperature increase limitations imposed on critical components, which can lead to decreased performance or in some cases a complete thermal runaway event. Battery pack design and material selection are major areas of investigation for improvements and future development in this field. From a materials standpoint, thermal conductivity is a key metric of consideration. Furthering this point, materials with anisotropic thermal behavior have shown great promise in their ability to reduce thermal runaway propagation in adjacent cells. Herein we discuss characterization methods for these novel materials, including application examples.

### 15.2. Thermal Stability of Different Cathode Ratios for Ternary Lithium-Ion Battery

ShuChi-Min*HaungWei-XiangDepartment of Safety, Health, and Environmental Engineering, National Yunlin University of Science and Technology*Correspondence: shucm@yuntech.edu.tw

**Abstract:** The energy density of LiNixCoyMnzO2 (NCM) can be effectively improved by increasing the Ni content ratio over 0.5 and the cut-off voltage over 4.5 V. Still, these materials suffer from high initial reversible capacity loss and low cycle life poor thermal stability. This study will compare the thermal stability of NCM cathode materials with different ratios. This study used differential scanning calorimetry (DSC) to test thermal stability. Model-free thermodynamic analysis simulation and apparent activation energy for different cathode materials through advanced kinetics and technology solution (AKTS). Thermogravimetric analysis (TGA) was used to analyze the progress of the stability of the cathode material to different temperatures, and Fourier transform infrared spectrometer (FT-IR) was used to analyze the gas composition released from the cracking of the cathode material. The DSC experimental results show that the NCM 811 100% SoC has the earliest exothermic onset temperature of 159.07 °C, while the NCM 523 50% SoC has an exothermic onset temperature of 225.68 °C. The NCM 811 100% SoC has the lowest apparent activation energy calculated by AKTS software, 99.0 kJ/mol. TGA observed that as the nickel content and charge state of the cathode material increased, the cathode material would be induced to crack at lower temperatures. The FT-IR results show that at a temperature of about 120 °C, all cathode materials have a tensile vibration with a functional group of 1866 cm^−1^ C=O, which also proves that the first stage of the weight loss reaction in the TG experiment is a solid electrolyte interface (SEI) or the decomposition of the electrolyte. The thermal stability of the ternary lithium-ion battery was investigated by the parameters obtained from the above experiments.

## 16. Session: General Posters

### 16.1. Quantification of Fiber Compositions in Nonwoven Fleece Materials

ZhangDongmeiReynolds American Inc
zhangd@rjrt.com

**Abstract:** Nonwoven fleece materials are widely used in pouch products such as tea bags and oral products in the food industry. Nonwoven fleece material is typically composed of a binder and various types of fibers. Rayon and polyester are two materials commonly used as fibers in oral product pouch fleece materials. A method was developed to analyze the fiber material and to quantify the inclusion level of each fiber type when used in a binary fiber mixture of rayon and polyester using thermal gravimetric analysis (TGA) and Fourier transform infrared (FT-IR) microspectroscopy. The identification of binder and fiber composition was performed by using FT-IR microspectroscopy to determine the suitable solvent for binder removal. TGA tests showed that the decomposition between 200 °C and 380 °C was unique to rayon. Therefore, the weight loss at this range is proportional to the percentage inclusion of rayon in a binary fiber mixture. A six-point calibration curve was established by mixing rayon fiber standard and polyester fiber standard in the range of 0% to 100% and analyzing the weight loss between 200 and 380 °C. Several commercial pouch fleece materials were quantified for percent inclusion of each type of fiber in a binary fiber mixture.

### 16.2. Method for Analyzing the Evolving Vapor Phase from Materials Subject to TGA Analysis

GanHarryReynoldsR. J.MoldoveanuSerbanReynoldsR. J.
ganh@rjrt.com

**Abstract:** Thermogravimetric analysis (TGA) is performed on a variety of samples, providing useful information. This information can be further extended by collecting the volatiles generated during the TGA analysis and performing a GC/MS analysis of them to determine their composition and relative level. For this purpose, a minor modification of an existing TGA instrument (Discovery TA 5500) was performed by attaching a fused silica capillary cooled in an ice bath to the TGA instrument where the volatiles generated can be collected. These volatiles are further analyzed by GC/MS either without any processing, or after derivatization, for example to form trimethylsilyl (TMS) derivatives. The procedure indicated as TGA/GC/MS is used in the present study to evaluate tobacco, reconstituted tobacco (recon), and several organic acids. The results allow the evaluation of different volatile compounds generated by heating tobacco and reconstituted tobacco. The study on organic acids evaluated their volatility and stability. For example, benzoic acid was transferred in volatile form during the TGA heating up to 340 °C without any significant decomposition, while other studied organic acids decomposed during the TGA heating up to that temperature.

### 16.3. Synthesis, Purification, and Characterization of N,N’-Di(1-naphthyl)-N,N’-diphenylbenzidine

SloanGilbert^1^*SzewczykSteven J.^1^McGhieAndrew^2^1MSE Dept. Univ. of PA2LRSM, University of PA*Correspondence: mcghie@lrsm.upenn.edu

**Abstract:***N,N’*-Di(1-naphthyl)-*N,N’*-diphenylbenzidine, NPB, is a large organic molecule (mw 588) of scientific interest as a material in OLED technology. Commercially, it was being offered at more than USD 500/gm, so we synthesized it ourselves (by GJS) using a simpler technique. A high purity is critical for optimum device performance, and many workers have used sublimation for purification, with mixed success. We used a modified Ullman synthesis, from 4,4′-diiodobiphenyl and 1-naphthyl-phenylamine, using finely divided copper as a catalyst, with no solvent, but a large excess of the amine reactant. The reaction is exothermic and reaches completion in a few minutes. The product was obtained as a gray-green solid, m.pt. 276-9 °C. Purification was achieved by zone melting, with 26 zone passes at 2 inches/hour in a Sloan McGowan Zone Refiner, to yield very fine, yellow-gray crystals. To obtain a fine, crystalline mass is unusual in an organic crystal that has been purified by zone melting and, in this case, is due to the fact that the liquid–crystal interface is actually a liquid–glass interface with subsequent nucleation of a crystalline phase in the glassy phase on cooling. DSC was used to study the thermodynamics and kinetics of this transition and to determine purity. The melting point by DSC had an onset at 279.5 °C (279, 280 °C quoted for 98% pure material). Additional chemical analysis was carried out using LC-MS. We believe this is one of the first studies on zone melting a glass-forming material that resulted in purification. A more complete report and DSC study will be presented at the conference.

### 16.4. Powder Flowability Study to Optimize Processing and Predict Final Product Performance

KanapathySandra*ReynaudSaraArkema*Correspondence: sandra.kanapathy@arkema.com

**Abstract:** The rheology of granular flow has recently been a topic of interest for additive manufacturing, 3D printing and laser sintering applications where the polymer is directly processed in powder form. In this work, we study the effect of particle physical properties, moisture and fillers on powder cohesion and flowability. In particular, we will discuss a combination of analytical and rheological testing to correlate powder structural properties with processing performance.

### 16.5. Practical Use of Temperature Modulated Differential Scanning Calorimetry for Thermal Properties Evaluation

HussainIstiakChemours*Correspondence: istiak.hussain@chemours.com

**Abstract:** Differential scanning calorimetry (DSC) is widely used to analyze the thermal properties of materials—for example, glass transition, melting point, enthalpy of fusion, heat capacity, etc. DSC measures the temperatures and heat flows associated with transitions in materials as a function of temperature or time in a controlled environment. However, these transitions could overlap in cases where multiple thermal phenomena occur in the same temperature range, which can lead to a misinterpretation of the analysis. Moreover, the measurement of heat capacity using standard DSC (ASTM E1269) usually requires a minimum of three experiments and yields an accuracy no better than ± 10%. Modulated DSC (MDSC) is an advancement over standard DSC that uses a sinusoidal temperature oscillation that separates the total heat flow into reversing and non-reversing components. This separation provides the ability to separate overlapping transitions, enhance interpretation of complex thermal curves, and measure the heat capacity in a single experiment with increased accuracy. This paper outlines the basis and the usefulness of the MDSC technique by means of examples and shows its value in the acquisition of information that standard DSC cannot readily tackle.

### 16.6. Photo-Thermal Writing on Co-Extruded Multilayer Optical Films for Data Storage Applications

MedinaSergio^1^ChaudhariNiket^1^NauferNusran^1^ShiyanovskayaIrina^1^*SingerKenneth^1^,^2^1Folio Photonics, Inc2Case Western Reserve Univ.*Correspondence: irina.shiyanovskaya@foliophotonics.com

**Abstract:** Multilayer polymer films are a promising approach for realizing high-capacity optical media with terabyte scale for applications in archival data storage. We are developing the next generation of optical data storage that is affordable, long-lived and energy efficient. Our technology is based on photo-thermal writing with short laser pulses on multilayer films. These films are made by co-extruding a polymer containing an organic dye that absorbs light and transforms it into heat. The different layers are produced by passing the dye-polymer mixture through a series of multipliers, which results in thin films with the optically active material, spaced out with a buffer layer of a passive polymer. Here, we report on the co-extrusion process, rheology and thermal properties of the polymers forming the multilayer media to fabricate highly uniform nanoscopic active layers. We discuss the threshold photo-thermal writing mechanisms, its critical factors and non-destructive readout of written data on multilayer films. Understanding the mechanisms permits the design of improved materials providing high capacity as well as read/write methods, thus defining our product offering. The multilayer optical data storage medium promises a high-performance entry into the data storage ecosystem, as its multilayer co-extrusion fabrication process is inherently low-cost and highly scalable.

## 17. Session: Student Poster

### 17.1. Fabrication of Electrospun Keratin-Silk Nanofibers with Tunable Structures and Properties

MazahrehJanineYildizKeremDurantFredrickCruzDavid Salas-de laHuXiaoRowan University
hu@rowan.edu

**Abstract:** Keratin is a protein biopolymer that is ubiquitous in nature, displaying several useful characteristics including a high stability attributed to its disulfide bonds. Keratins have unique structures and properties, involving a specific peptide sequence and hydrophilic nature that can promote cell attachment and growth, allowing this fibrous protein to have a high function in several medical applications. Keratins located in protofibrils within the structure of a wool fiber are arranged in a coiled form, which link with microfibrils that join the macrofibrils at inter- and intramolecular points through hydrogen, ionic, and disulfide linkages. These bonds are responsible for the classification of keratin as a stable biomaterial, making it practical for various applications, such as wound healing technology. In the present research, keratin was extracted from wool and combined in several ratios with silk fibroin in formic acid solution with 4% CaCl_2_ for electrospinning. Characterization tests such as Fourier-transform infrared spectroscopy (FTIR), differential scanning calorimetry (DSC), scanning electron microscopy (SEM), and thermogravimetric analysis (TGA) were performed on the resulting nanofibers. Through SEM, it was confirmed that all ratio samples produced homogeneous nanofibers, except for pure keratin, which exhibited strong beading during the electrospinning process. An increase in keratin resulted in finer and stronger nanofibers, with the 50:50 ratio producing the most enhanced nanofibers. FTIR analysis suggested that electrospinning these materials did not change the strong secondary structure of each material. DSC and TGA confirmed the thermal stability of keratin–silk biomaterials.

### 17.2. Effects of LiCl on the Thermal Properties of Polyzwitterions

ThomasJohnCebePeggyBiswasYajnaseniAsatekinAyseTufts University
lang.wei@dupont.com

**Abstract:** Polyzwitterion (PZI) and salt complexes can be used in solid polymer electrolyte applications because of their ionic conductivity, thermal stability, and mechanical stability [1]. In this study, we investigate changes in the solid state properties of three PZI/LiCl complexes, poly(sulfobetaine methacrylate), PSBMA, poly(sulfobetaine acrylate), PSBA, and poly(ethyl sulfobetaine methacrylate). These complexes were created by dissolving LiCl at various weight percentages of 1–20% wt LiCl. Changes in the solid-state heat capacity, glass transition temperature (Tg), and change in heat capacity at the glass transition (Δcp) were measured using temperature-modulated differential scanning calorimetry (TMDSC). PZIs and LiCl are both hydrophilic and hygroscopic. The presence of water plasticizes the glass transition, moving it to lower temperatures. To ensure that the sample is properly dried, a ratchet method is employed. Here, the sample is heated and cooled at 20 °C/min to successively higher temperatures to slowly eject both surface-bound and molecularly bound water. Each subsequent heating reduces the plasticization effect until the final dry state glass transition is achieved. Thermogravimetric analysis (TGA) is used to measure changes in the onset of thermal degradation (Td) as a function of salt content. PZIs have two main stages of degradation: the first is the breakdown of the side chain (Td1), while the second is the breakdown of the backbone (Td2). Through TGA, it is seen that as the salt content increases, there is a decrease in the onset of Td1 but an increase in the onset of Td2. Finally, through TMDSC it is shown that as the salt content increases, the Tg decreases and the Δcp increases due to the breaking of the intra- and inter-molecular crosslinks by association of the LiCl with the zwitterionic moiety.

**References** 

1. Cardoso, J., et al., Synthesis and characterization of zwitterionic polymers with a flexible lateral chain. The Journal of Physical Chemistry C, 2010. 114(33): p. 14261-14268.”

### 17.3. Thermal and Raman Spectroscopic Analysis of Electro-Active (Polar) Poly(vinylidene fluoride)

JayasekaraAnujaCebePeggyTufts University
anuja.jayasekara@tufts.edu

**Abstract:** Poly(vinylidene fluoride), PVDF, is a semi-crystalline polymer with three common crystalline polymorphs: the alpha, beta, and gamma phases [1]. The beta and gamma phases are known as electro-active (polar) phases of PVDF because of the strong dipole moments arising due to their structural conformations [2]. Difficulty in isolating beta-phase and gamma-phase crystals in PVDF leads to complexities in estimating their respective crystalline fraction and as well as in determining their equilibrium heats of fusion. We used two different preparation methods to obtain, predominantly, either the beta phase or the gamma phase of PVDF. Electrospinning is known to produce dominantly beta-phase crystals in PVDF because the polymer is stretched by the simultaneous application of electrical and mechanical forces [3]. γ-phase dominant PVDF films were prepared from solutions using dimethylacetamide as the solvent [2]. The PVDF fibers and films were analyzed using differential scanning calorimetry, Fourier transform infrared spectroscopy, and Raman spectroscopy. In recent years, the intensity analysis of the peaks in Raman spectra has been widely used in estimating the degree of crystallinity in polymers [4–6]. Temperature-controlled Raman analysis was carried out to identify the peaks corresponding to beta- and gamma-phase PVDF. In this study, estimates of the relative degrees of crystallinity of beta- and gamma-phase PVDF and the equilibrium heat of fusion of these polar phases of PVDF are presented.

**References** 

1. Cozza ES et al., On the electrospinning of PVDF: influence of the experimental conditions on the nanofiber properties. Polym. Int. 2013; 62(1): 41-48.
2. Lovinger AJ, Poly(Vinylidene Fluoride), in Developments in Crystalline Polymers—1, D.C. Bassett, Editor. 1982; Springer Netherlands: Dordrecht: 195-273.
3. Lei T et al., Electrospinning-induced preferred dipole orientation in PVDF fibers. J. of Mater. Sci. 2015; 50(12): 4342-4347.
4. Agarwal UP, RS Reiner, and SA Ralph, Cellulose I crystallinity determination using FT–Raman spectroscopy: univariate and multivariate methods. Cellulose. 2010; 17(4): 721-733.
5. Kotula AP, CR Snyder, and KB Migler, Determining conformational order and crystallinity in polycaprolactone via Raman spectroscopy. Polym. 2017; 117: 1-10.
6. Nielsen AS, D Batchelder, and R Pyrz, Estimation of crystallinity of isotactic polypropylene using Raman spectroscopy. Polym. 2002; 43(9): 2671-2676.”

### 17.4. Crystallization of Poly(ethylene oxide) Side Chain-Bearing Star Bottle Brushes

FurnerCarl^1^*WilkJeffrey^1^KellyMichael^2^KentEthan^2^ZhaoBin^2^LiChristopher^1^1Drexel University2University of Tennessee, Knoxville*Correspondence: ctf46@drexel.edu

**Abstract:** Molecular bottlebrush copolymers have a unique polymer architecture and present opportunities in biomedical, photonic, surface functionalization, and other fields [1,2]. Bottlebrush copolymers can be formed through a click chemistry reaction between the backbone polymer and the side chains, which can be controlled to produce copolymers of different backbone grafting density [3]. A feature of this synthetic route is the ability to form multi-armed star bottlebrushes through the addition of one or more backbone arms. Previously, our group investigated the effect of grafting density on side chain crystallization in linear bottlebrush copolymers and have shown that different brush architectures significantly influence aspects of side-chain crystallization. We seek to build upon this work through the investigation of three-arm star brush polymers. A series of poly(2-hydroxyethyl methacrylate)-*g*-poly(ethylene oxide) (PHEMA-g-PEO) star brush polymers were synthesized through a copper-catalyzed azide-aklyne cycloaddition process. A series of samples with different grafting densities were developed by varying the feed ratio of backbone monomer PHEMA units to side chain PEO units. A variety of differential scanning calorimetry experiments were designed to investigate the effect of the addition of a ternary arm to a pHEMA-g-PEO star bottlebrush architecture at different grafting densities. Non-isothermal measurements show that the crystallization of the side chains of star brush polymers is hindered, contains an architecture dependence, and shows marked differences from both the linear PEO and linear brush PEO. A thermal fractionalization protocol was developed to investigate the retention of crystalline nuclei after the nominal melting point, showing that DII memory is retained and the isotropic melting point increases with increasing grafting density. We envision that this classical characterization can promote the development of processing and applications of these unique polymers.

**References** 

1. A. L. Liberman-Martin, C. K. Chu, and R. H. Grubbs, “Application of Bottlebrush Block Copolymers as Photonic Crystals,” Macromolecular rapid communications., vol. 38, no. 13, p. 1700058–n/a, 2017, doi: 10.1002/marc.201700058.
2. R. Verduzco, X. Li, S. L. Pesek, and G. E. Stein, “Structure, function, self-assembly, and applications of bottlebrush copolymers,” Chemical Society reviews, vol. 44, no. 8, pp. 245–242, 2015, doi: 10.1039/c4cs00329b.
3. T. Wu, X. Leng, Y. Wang, Z. Wei, and Y. Li, “Linear- and star-brush poly(ethylene glycol)s: Synthesis and architecture-dependent crystallization behavior,” Polymer (Guilford), vol. 202, p. 122661–, 2020, doi: 10.1016/j.polymer.2020.122661.

### 17.5. Analysis of Biaxially Cold Rolled Poly(phenylene sulfide) Using Temperature Modulated Differential Scanning Calorimetry and Broadband Dielectric Spectroscopy

McMullenNathanRuiG.BaselB.ZhuL.WnekG. E.Case Western Reserve*Correspondence: nam77@case.edu

**Abstract:** Temperature-modulated differential scanning calorimetry has been used to study the crystallization behavior in poly(phenlyene sulfide) (PPS) biaxially oriented by roll milling at room temperature. The total heat flow trace reveals an exothermic peak associated with cold crystallization at about 125 °C and a glass transition temperature of 92 °C for melt crystallized PPS. After cold rolling, the cold crystallization peak appeared to shift to temperatures in the vicinity of the glass transition temperature. The non-reversing signal obtained from deconvolution of the total heat flow trace confirms that the cold crystallization peak had shifted to a lower temperature. The reversing signal was used to conclude that the glass transition temperature associated with the mobile amorphous fraction was unchanged after orientation, and that the rolled material is subject to enthalpy relaxation upon heating beyond Tg. Due to the overlapping cold crystallization peak and glass transition temperature after cold rolling in the total heat flow trace, this analysis would not have been possible with DSC in the standard mode. Broadband dielectric spectroscopy (BDS) was used to further investigate the crystallization behavior of roll-oriented PPS. In a film of melt-processed PPS, a frequency variant peak associated with the glass transition was observed in the loss component of the complex relative permittivity. A frequency invariant peak was also observed at the cold crystallization temperature (around 120 °C), but only at low frequencies. After roll orientation, the frequency variant glass transition peak was still present, but not the frequency invariant cold crystallization peak. A shoulder also appeared in the physical aging range after rolling, but was only present at low frequencies.
**Funding:** This work was made possible by the generous support from the US Army Research Lab (ARL) via Contract # W911NF2020155.

